# Tripterygium glycosides for safely controlling disease activity in systemic lupus erythematosus: a systematic review with meta-analysis and trial sequential analysis

**DOI:** 10.3389/fphar.2023.1207385

**Published:** 2023-08-04

**Authors:** Yifan Chen, Liuding Wang, Nannan Li, Caiyun Zhou

**Affiliations:** Xiyuan Hospital, China Academy of Chinese Medical Sciences, Beijing, China

**Keywords:** tripterygium glycosides, systemic lupus erythematosus, lupus activity, systematic review, meta-analysis

## Abstract

**Background:** Tripterygium glycosides have been used to treat systemic lupus erythematosus (SLE) for a long time, showing the effects of immune regulation. We aimed to evaluate the benefits and risks of Tripterygium Glycosides Tablets (TGT) for patients with SLE.

**Methods:** We searched electronic databases and clinical trial registries for relevant randomized controlled trials (RCTs). We identified eligible RCTs and assessed risk of bias. We conducted a meta-analysis to estimate the pooled effects. The Trial Sequential Analysis (TSA) 0.9.5.10 software was used to verify the reliability of the results.

**Results:** Eight RCTs encompassing 538 patients with SLE were included. TGT combined with conventional treatments (CTs) was superior to CTs alone in reducing lupus activity (*MD* = −1.66, 95% *CI* = −2.07 to −1.26, *p* < 0.00001, low-certainty evidence) and improving overall response rate (ORR) (*RR* = 1.21, 95% *CI* = 1.11 to 1.32, *p* < 0.0001, moderate-certainty evidence). The robustness of the results was confirmed by TSA. Regarding safety, there was no statistical difference in the overall incidence of adverse reactions between the two groups.

**Conclusion:** In patients with SLE, TGT might safely reduce disease activity. However, further high-quality studies are needed to firmly establish the clinical efficacy of TGT.

**Systematic Review Registration:** https://www.crd.york.ac.uk/prospero/display_record.php?ID=CRD42022300474; Identifier: CRD42022300474.

## Introduction

Systemic lupus erythematosus (SLE) is a chronic immune-mediated disease characterized by multiple organ damage, leading to cutaneous, joint, and systemic manifestations (Piga and Arnaud, 2021). Fatal complications of SLE, such as neuropsychiatric lupus erythematosus with obscure pathogenesis and various other symptoms, pose particular challenges for the management of patients with lupus (Zhang et al., 2021). An increased risk of developing severe comorbidities, including cardiovascular diseases, pulmonary hypertension, infections, and kidney failure, is associated with persistent lupus activity (Mu et al., 2018). Additionally, the relapsing-remitting rate in patients with SLE was approximately 70% according to a 10-year follow-up study (Tselios et al., 2019). Consequently, SLE poses a substantial burden to both patients and caregivers (Jönsen et al., 2015).

Pharmacotherapies predominantly recommend hydroxychloroquine, glucocorticoids, and immunosuppressants (Tunnicliffe et al., 2015). The emergence of these available therapies has improved the prognosis of SLE (Mak et al., 2012; Lisnevskaia et al., 2014), however, numerous toxicities of current regimens have been identified (including fertility failure, birth defects, and infections) (Doyno et al., 2021). Benefits of hydroxychloroquine during pregnancy are generally considered to outweigh the teratogenic risk (Bérard et al., 2021; Huybrechts et al., 2021). Nevertheless, retinal toxicity remains a worrying complication in patients treated with antimalarial drugs (Mukwikwi et al., 2020). Regarding prescribed immunosuppressants, a higher cumulative cyclophosphamide dose is associated with a greater possibility of premature ovarian failure and congenital malformations (Rengasamy, 2017; Sen et al., 2021). Despite their powerful capability to induce immunosuppression, corticosteroids are responsible for the majority of infections, permanent organ damage, and premature death in patients with lupus (Thamer et al., 2009; Apostolopoulos and Morand, 2016). Hence, a novel alternative therapy, remitting the disease activity without increased damage attributable to side-effects, needs to be developed (Lazar and Kahlenberg, 2023).

Research and development of traditional Chinese medicines (TCMs) to decrease lupus activity is promising, and recent evaluations of clinical evidence indicated that several Chinese botanical drugs, including Qinghao Biejia decoction, Zhibo Dihuang pill, and total glucosides of paeony, might reduce the disease activity and cumulative dose of steroids used (Dai et al., 2020; Li et al., 2021; Wang et al., 2021; Chen et al., 2022). *Tripterygium wilfordii* Hook F (*Tw*HF) (Celastraceae; tripterygium), a widely used TCM plant against autoimmune diseases in China (Li et al., 2015; Li et al., 2021; Lin et al., 2021), is the only Chinese botanical drug mentioned in the 2020 Chinese guidelines for the diagnosis and treatment of SLE (Chinese Rheumatology Association et al., 2020). Tripterygium glycosides (TG) are fat-soluble extracts from dried roots of *Tw*HF. Tripterygium Glycosides Tablets (TGT) are representative *Tw*HF-based agents that are included in the 2019 edition of the Medicine Catalog for National Basic Medical Insurance, Injury Insurance, and Maternity Insurance, and have been extensively studied in the treatment of SLE. TGT consist of diterpenoids, triterpenoids, and alkaloids. Triptolide (TP) has proven to be not only the most active compound of TGT (Ma et al., 2007), but also the major contributor to TGT toxicity *in vivo*, which induces hepatotoxicity, nephrotoxicity, and testicular toxicity (Shen et al., 2014; Li et al., 2015; Xiong et al., 2019; Ge et al., 2023). In addition to male reproductive toxicity, it also has ovarian toxicity (Qiu et al., 2011; Liu et al., 2017). Both TP and wilforlide A (WA) are the quality control markers of TGT. The recommended content of TP in each tablet of TGT should be less than 10 mcg, while that of WA should be greater than 10 mcg (National Medical Products Administration of China, 2003). Due to the complexity of the aforementioned compounds, it is evident that a wide range of targets and signaling pathways have been involved in the pharmacological mechanisms of TGT. For example, NLRC3 may be a potential target for TGT treatment of bone and joint complications related to SLE (Xu et al., 2023). Upon investigation of its immunosuppressive effects on adjuvant-induced arthritis (AIA) rats and collagen-induced arthritis (CIA) mice, researchers have found that down-regulating T helper 17 cells (Th17) and up-regulating T regulatory cells (Treg) may be the potential mechanism of action (Wang et al., 2008; Astry et al., 2015). Recently, a network-pharmacological study uncovered the key targets affected by TG during SLE treatment (Xiao et al., 2022).

The unpredictable flare and clinical heterogeneity of SLE, as well as the current toxicities of treatment regimens, indicate that the identification of novel, safe, and effective alternative drugs is of great importance (Durcan et al., 2019). Considering the perniciousness of SLE and immune effects of TGT, this study aimed to evaluate the efficacy and safety of TGT for the treatment of patients with SLE.

## Methods

### Protocol registration

The protocol was registered (PROSPERO: CRD42022300474), and the systematic review and meta-analysis was reported according to the Preferred Reporting Items for Systematic Reviews and Meta-Analyses (PRISMA).

### Data sources and searches

We searched the following electronic databases from their inception to November 21, 2022: PubMed, Embase, Cochrane Central Registry of Controlled Trials (CENTRAL), China National Knowledge Infrastructure (CNKI), Wanfang Database, SinoMed, and the China Science Technology Journal Database. Both the ClinicalTrials (http://www.clinicaltrials.gov) and Chinese Clinical Trial Registry (http://www.chictr.org.cn/) were also searched. All search strategies are presented in Supplementary Table S1.

On April 17, 2023, we updated the search of all the abovementioned databases.

### Inclusion criteria

We included randomized controlled trials (RCTs), published in Chinese or English, in patients diagnosed with SLE according to the American College of Rheumatology revised criteria for the classification of SLE (Hochberg, 1997). In these trials, subjects received TGT alone or combined with conventional treatments (CTs) in the intervention groups, and patients received the same CTs alone or combined with other positive drugs in the control groups. Hydroxychloroquine, glucocorticoids, immunosuppressive drugs, and biological agents were included as CTs according to the European League Against Rheumatism recommendations for the management of SLE (Fanouriakis et al., 2019). Primary outcomes were SLE Disease Activity Index (SLEDAI), SLE responder index 4 (SRI-4) and overall response rate (ORR). The SLEDAI included the SLEDAI-2K (Gladman et al., 2002) and the original SLEDAI (Bombardier et al., 1992). Secondary outcomes included the quality of life, 24-h urine protein, anti-double-stranded DNA (anti-dsDNA), immunoglobulins, and complement proteins. Safety outcome was the incidence of adverse reactions.

### Exclusion criteria

The following trials were excluded: 1) trials using other *Tw*HF preparations or TCMs in the intervention or control groups; 2) trials repeatedly reporting the same data, with the suspicion of duplicate publication; 3) trials reporting results that were inconsistent with the conclusions drawn, with the suspicion of academic fraud; 4) trials without reporting important information, such as course of treatment and dosage of TGT; and 5) trials focusing on special populations, such as SLE in children and adolescents.

### Study selection

Two authors (YFC and LDW) selected studies independently. All retrieved records were imported into NoteExpress 3.2 and the duplicates were removed. The titles and abstracts of the remaining records were screened, and the full texts of potentially relevant studies were subsequently screened. Disagreements were resolved through a discussion with a third author (NNL).

### Data extraction

Two authors (YFC and LDW) extracted data using a prespecified form, cross-checked the accuracy of the extractions, and resolved disagreements through discussion with a third author (NNL). The following data was collected: authors, publication year, sample sizes, methodological quality (random sequence, allocation concealment, and details of blinding), baseline characteristics of participants (age, gender, SLEDAI, and duration of treatment), frequency and dose of drug administration, and outcomes.

### Risk of bias assessment

Two authors (YFC and LDW) independently assessed the risk of bias in the included trials. Using the Cochrane risk of bias tool 2.0 (Sterne et al., 2019), the following five domains were evaluated: randomization process, deviations from intended interventions, missing outcome data, outcome measurements, and selective reporting. Any disagreements were resolved by consulting a third author (NNL).

### Data analysis

Statistical analyses were performed using the RevMan 5.4 software. Continuous outcomes (SLEDAI score and laboratory findings) were assessed using the weighted mean difference (*WMD*) or standardized mean difference (*SMD*), and dichotomous outcomes (SRI-4, overall response rate, and incidence of adverse reactions) were assessed using the risk ratio (*RR*). All analytical tools were demonstrated with effect size and 95% confidence intervals (*CI*). A fixed-effects model was applied once the *I* ^2^ was ≤50%. Otherwise, a random-effects model was used to perform the data analysis. To address the clinical heterogeneity, subgroup analyses based on the course of treatment were performed. To explore the sources of statistical heterogeneity, sensitivity analyses, omitting one trial at a time, were performed. If the change in statistical heterogeneity was significant after trials were removed, the full texts further were re-visited. To estimate the required information size and evaluate the robustness of the results, trial sequential analysis (TSA) was performed using the TSA 0.9.5.10 software. To assess small-study effects, Begg’s rank correlation and Egger’s linear regression tests were performed to detect the publication bias.

#### Certainty assessment

Two authors (YFC and LDW) independently evaluated the certainty of evidence in accordance with the Grading of Recommendations Assessment, Development and Evaluation (GRADE) approach (Balshem et al., 2011) and the level of evidence was judged as “high”, “moderate”, “low”, or “very low”.

## Results

### Study selection

**FIGURE 1:**
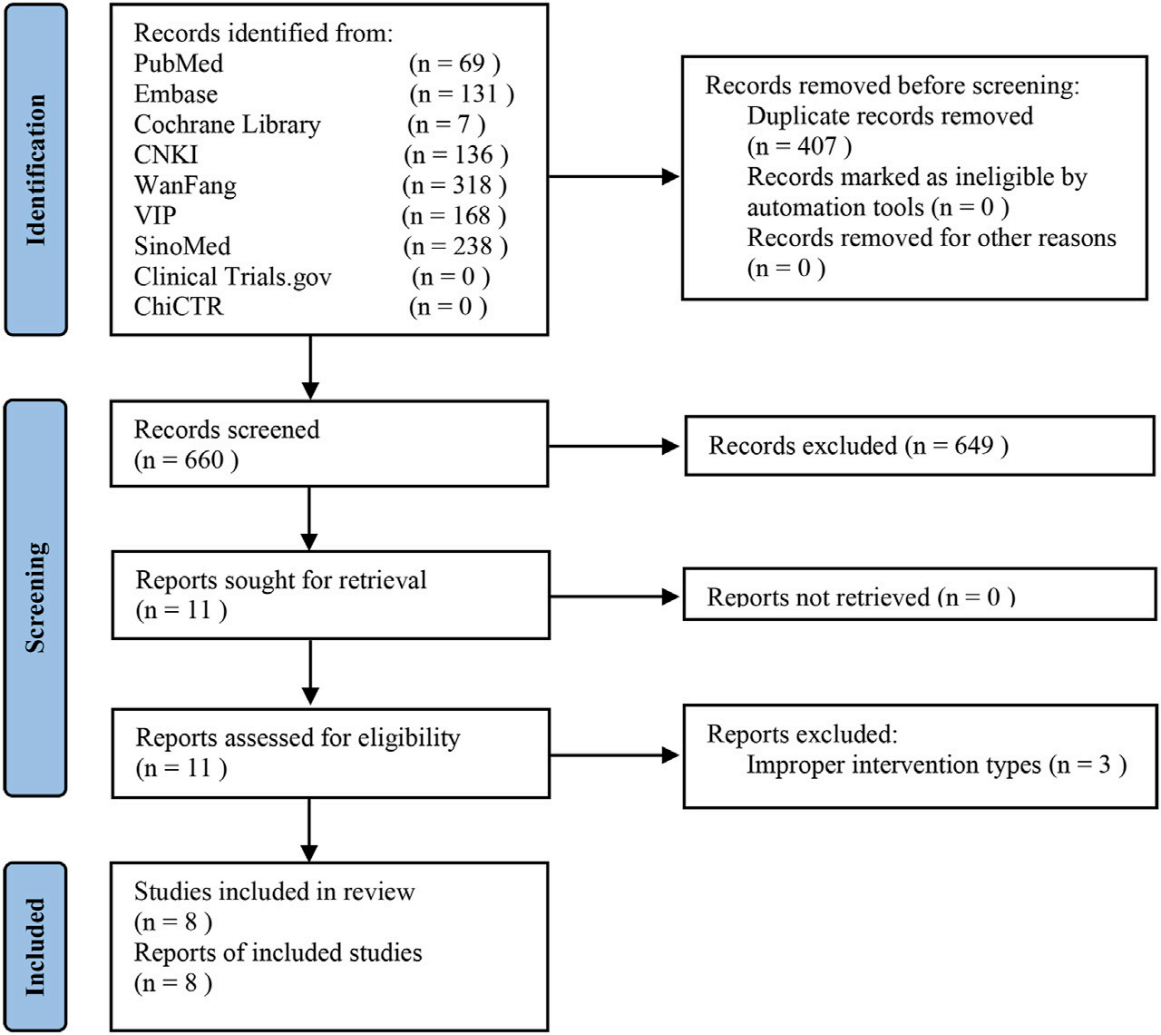
PRISMA flow diagram for identification of studies.

Figure 1 presents how we identified eight eligible RCTs (Liu et al., 2014; An and Fang, 2015; Li and Luo, 2015; Chen, 2018; Li et al., 2018; Wang, 2018; Zhang, 2018; Wang, 2022). Our initial searches yielded 1067 records. After removing 407 duplicates, we screened titles and abstracts, and then excluded 649 ineligible records. Eleven trials were selected for full-text evaluation. A list of three trials that appeared to meet the inclusion criteria but were excluded are reported in Supplementary Table S2 along with citations and reasons for exclusion.

### Study characteristics

Eight RCTs involving 538 participants included 272 participants in the intervention groups and 266 in the control groups. These included articles were published from 2014 to 2022. Sample sizes of the trials ranged from 30 to 100 participants. One trial (Liu et al., 2014) reported a comparison of TGT plus glucocorticoid and methotrexate (MTX) plus glucocorticoid, whereas other trials reported a comparison of TGT plus CTs and CTs alone. In three trials, participants were treated with 20 mg TGT three times daily for 1 month (An and Fang, 2015) or 6 months (Liu et al., 2014; Li et al., 2018); in the other trials, participants were treated with 1.0–1.5 mg/kg/day TGT for 3 months or 4 months. Additionally, the SLEDAI score was reported by six trials (Liu et al., 2014; An and Fang, 2015; Chen, 2018; Li et al., 2018; Zhang, 2018; Wang, 2022). The details of sex, age, disease activity, and CTs in each trial are summarized in Table 1. The source, quality control, and chemical characteristics of TGT used in the included trials are presented in Supplementary Table S3.

**TABLE 1:** Basic characteristics of included studies.

Study	Sample size		Male/Female		Age/(year)		SLEDAI		Intervention		Treatment duration	Outcomes reported
	T	C	T	C	T	C	T	C	T	C		
Liu et al. (2014)	40	39	8/71		18–70	18–70	12.8 ± 4.15	12.6 ± 5.01	TGT 20 mg three times daily plus CTs (CTs: GC 0.5 mg/kg/day for 4 weeks, and then reduced it until 10 mg/day)	MTX 10 mg weekly plus CTs	6 months	1) 2) 3) 5) 7)
An and Fang (2015)	15	15	0/15	0/15	17–50 (34.7 ± 8.5)	17–50 (34.7 ± 8.5)	17.6 ± 1.7	17.7 ± 1.4	TGT 20 mg three times daily plus CTs (CTs: GC 0.8–1 mg/kg/day, and then reduced it according to the condition)	CTs	1 month	1) 3)
Li and Luo (2015)	44	43	9/78		17–50 (28.91 ± 9.82)	17–50 (28.91 ± 9.82)	Moderate activity		TGT 1.0–1.5 mg/kg/day three times daily plus CTs (CTs: GC 20 mg three times daily)	CTs	3 months	2) 3) 4) 5)
Chen (2018)	41	41	9/32	10/31	15–64 (31.08 ± 3.72)	16–68 (33.51 ± 3.96)	9.70 ± 2.57	9.93 ± 2.18	TGT 1.0–1.5 mg/kg/day three times daily plus CTs (CTs: GC 0.2–1.0 mg/kg/day, and then reduced it according to the condition; HCQ 0.2 mg twice daily for 2 weeks, and then adjusted it to 0.2–0.4 mg/day)	CTs	12 weeks	1) 2) 4) 5) 6) 7)
Li et al. (2018)	50	50	15/35	18/32	22–58 (34.2 ± 6.80)	22–63 (36.4 ± 6.10)	14.81 ± 4.14	14.63 ± 5.10	TGT 20 mg three times daily plus CTs (CTs: GC 0.5 mg/kg/day for a month, and then reduced it until 10 mg/day)	CTs	6 months	1) 2) 7)
Wang (2018)	40	40	13/27	12/28	19–55 (35 ± 6.7)	17–54 (34 ± 6.7)	Moderate activity		TGT 1.0–1.5 mg/kg/day three times daily plus CTs (CTs: GC 20 mg three times daily, NSAIDs, immunosuppressants)	CTs	3 months	2)
Zhang (2018)	15	15	/	/	31.07 ± 4.54	30.53 ± 4.29	4.8 ± 3.16	4.8 ± 2.67	TGT 1.0 mg/kg/day three times daily plus CTs (CTs: GC 0.5–1.0 mg/kg/day, and then reduced it according to the condition)	CTs	3 months	1)
Wang (2022)	27	23	10/17	9/14	21–38 (30.42 ± 3.62)	20–38 (30.87 ± 4.13)	11.35 ± 2.15	10.72 ± 2.79	TGT 1.0–1.5 mg/kg/day three times daily plus CTs (CTs: GC 45 mg daily, and then reduced it according to the condition)	CTs	4 months	1) 2)

C, control group; CTs, conventional treatments; T, intervention group; TGT, tripterygium glycoside tablets; GC, glucocorticoid; HCQ, hydroxychloroquine sulfate; MTX, methotrexate; NSAIDs, non-steroidal anti-inflammatory drugs.

Outcomes: 1) SLEDAI, score; 2) overall response rate; 3) 24-h urine protein; 4) anti-dsDNA; 5) complement proteins C3 and C4; 6) IgG; 7) adverse reactions.

### Assessment of risk of bias

**FIGURE 2:**
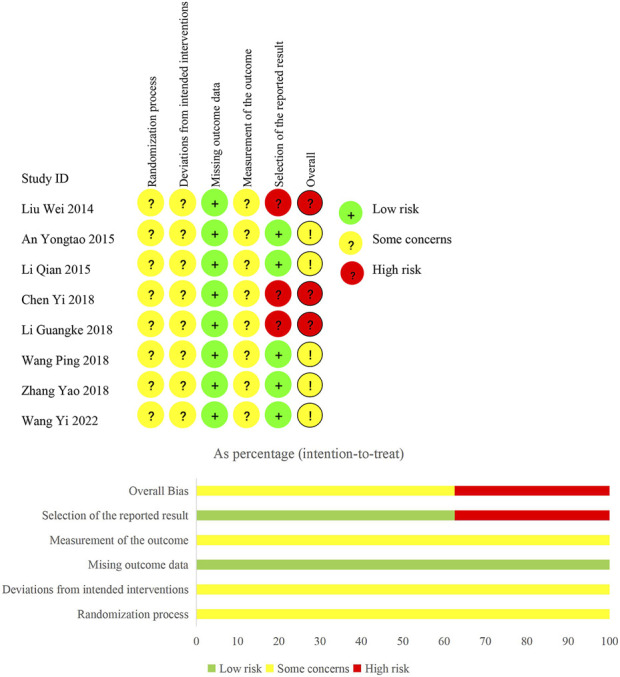
Risk of bias of included studies.

We judged the overall bias of three trials as “high risk of bias” and the overall bias of other trials as “some concerns.” The results are shown in Figure 2.

For random sequence generation, three trials (An and Fang, 2015; Chen, 2018; Li et al., 2018) used a random number table, one trial (Zhang, 2018) only mentioned the use of stratified random sampling, and four trials (Liu et al., 2014; Li and Luo, 2015; Wang, 2018; Wang, 2022) lacked the description of random sequence generation. For allocation concealment, none of the included trials reported the related information. Considering the insufficient information, we judged the risk of bias arising from the randomization process as “some concerns” in all trials. We failed to speculate the deviations from intended interventions and the bias in measurement, since information regarding blinding was unavailable. Therefore, we judged the risk of bias in these domains as “some concerns” in all trials. We judged the risk of bias due to missing outcome data as “low risk of bias” in all trials, because all outcome data was available. Additionally, although all trials completely reported the outcomes, three trials (Liu et al., 2014; Chen, 2018; Li et al., 2018) reporting adverse reactions lacked a specific description of the relationship between adverse reactions and medication. Hence, we judged the risk of selection bias as “high risk of bias” in the three trials and “low risk of bias” in the other trials.

### Primary outcomes

**FIGURE 3:**
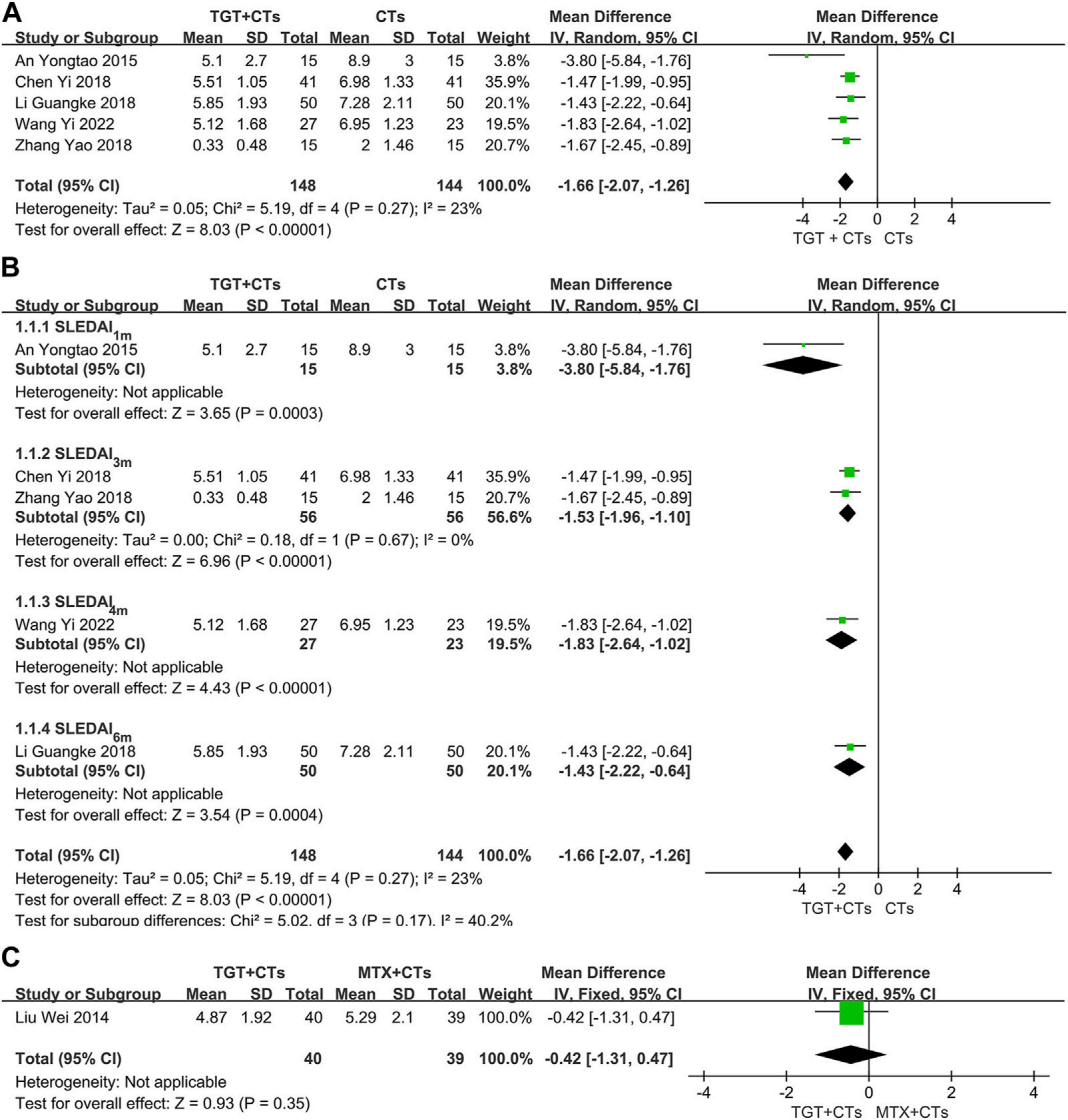
Forest plot of the SLEDAI score (*TGT + CTs vs*. *CTs*) **(A)**, subgroup analysis of the SLEDAI score (*TGT + CTs vs*. *CTs*) **(B)**, and SLEDAI score (*TGT + CTs vs. MTX + CTs*) **(C)**.

The SLEDAI score was reported in six trials after different durations of TGT administration, including 1 month (An and Fang, 2015), 3 months (Chen, 2018; Zhang, 2018), 4 months (Wang, 2022), and 6 months (Liu et al., 2014; Li et al., 2018). None of them reported the version of SLEDAI. When comparing TGT plus CTs *versus* CTs alone, the result found that TGT was associated with a statistical reduction in SLEDAI (*MD*_SLEDAI_ = −1.66, 95% *CI* = −2.07 to −1.26, *p* < 0.00001; Figure 3A). Despite the nonsignificant heterogeneity (*I* ^2^ = 23%), a random-effects model was employed due to different durations of treatment. In addition, a subgroup analysis was carried out based on the treatment durations to further determine the difference between short- and long-term efficacy of TGT against SLE. The subgroup analysis indicated that the most significant reduction was in the 1 month subgroup (*MD*_SLEDAI-1m_ = −3.80, 95% *CI* = −5.84 to −1.76, *p* = 0.0003; *MD*_SLEDAI-3m_ = −1.53, 95% *CI* = −1.96 to −1.10, *p* < 0.00001; *MD*_SLEDAI-4m_ = −1.83, 95% *CI* = −2.64 to −1.02, *p* < 0.00001; *MD*_SLEDAI-6m_ = −1.43, 95% CI = −2.22 to −0.64, *p* = 0.0004; Figure 3B). When comparing TGT *versus* MTX, one trial (Liu et al., 2014) demonstrated insignificant difference in decreased SLEDAI score between two groups (*MD* = −0.42, 95% *CI* = −1.31 to 0.47, *p* = 0.35; Figure 3C), which partially indicated that the effects of 20 mg TGT three times daily may be equivalent to that of 10 mg MTX weekly for SLE treatment.

**FIGURE 4:**
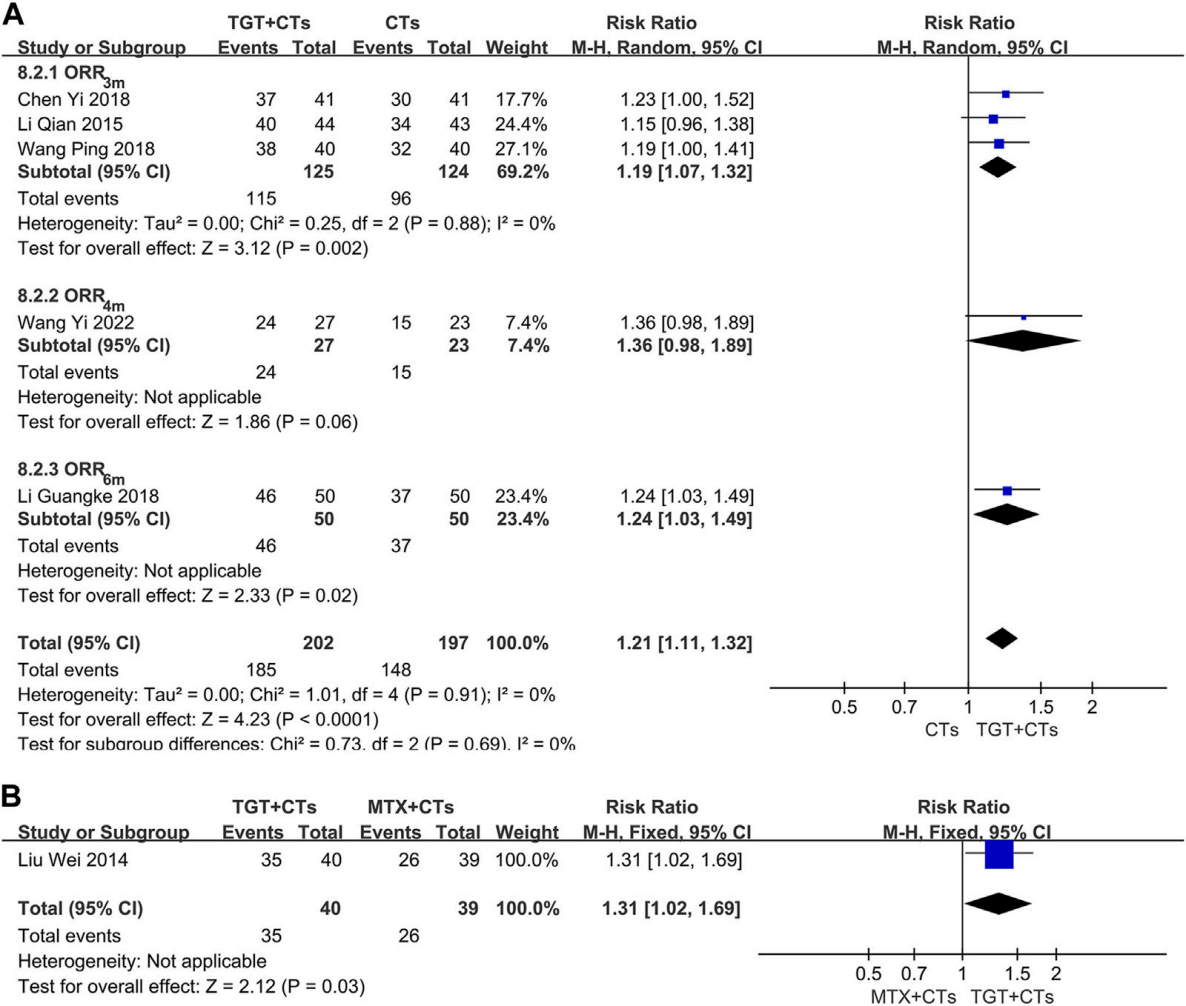
Forest plot of the overall response rate (*TGT + CTs vs. CTs*) **(A)** and overall response rate (*TGT + CTs vs. MTX + CTs*) **(B)**.

**FIGURE 5:**
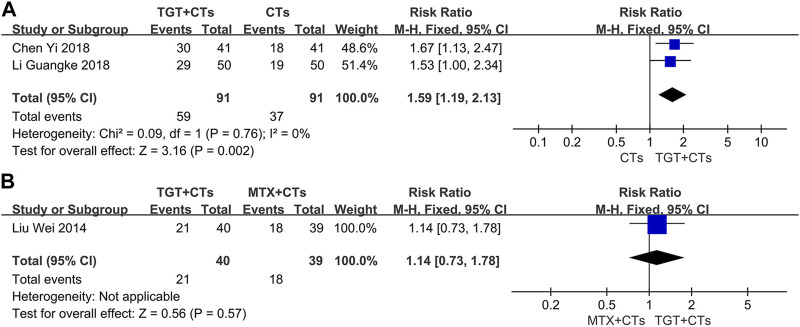
Forest plot of the significant response rate (*TGT + CTs vs. CTs*) **(A)** and significant response rate (*TGT + CTs vs. MTX + CTs*) **(B)**.

None of the included trials reported the SRI-4, a composite index requiring full improvement of SLE (Luijten et al., 2012), however, six trials (Liu et al., 2014; Li and Luo, 2015; Chen, 2018; Li et al., 2018; Wang, 2018; Wang, 2022) reported ORR, which is a composite index widely used in China. There was a significant difference in ORR between the TGT groups and the control groups (*RR*_ORR-3m_ = 1.19, 95% *CI* = 1.07 to 1.32, *p* = 0.002; *RR*_ORR-4m_ = 1.36, 95% *CI* = 0.98 to 1.89, *p* = 0.06; *RR*_ORR-6m_ = 1.24, 95% *CI* = 1.03 to 1.49, *p* = 0.02; *RR*_MTX_ = 1.31, 95% *CI* = 1.02 to 1.69, *p* = 0.03; Figure 4). Two evaluation criteria for ORR in the selected trials were used, including one focusing on the improvement of laboratory indicators and one focusing on the disappearance of symptoms. In the two trials that used the former criterion (Li and Luo, 2015; Wang, 2018), the significant response was defined as a platelet count reaching 100×10^9^/L without bleeding and normal levels of urine protein, anti-dsDNA, C3, and C4; in the other three trials that used the latter criterion (Liu et al., 2014; Chen, 2018; Li et al., 2018), the definition of significant response included the complete disappearance of symptoms. The significant response rate is more conservative, because the ORR contains a risk of exaggerating the real efficacy of drugs. Moreover, the significant response rate in the three trials, approximating a proportion of patients with lupus with disappearance of all symptoms and no new severe disease activity, has an advantage of detecting full improvement and no worsening, the result of which may have the same/similar significance as that of SRI. Consequently, we further performed a data analysis of this outcome. The results found that TGT plus CTs statistically improved the significant response rate compared with CTs alone (*RR* = 1.59, 95% *CI* = 1.19 to 2.13, *p* = 0.002; Figure 5A), whereas there was no difference in the significant response rate between the TGT group and the MTX group (*RR* = 1.14, 95% *CI* = 0.73 to 1.78, *p* = 0.57; Figure 5B).

Considering the clinical heterogeneity of CTs and TGT dosage, subgroup analyses were performed separately according to different chemical drugs such as hydroxychloroquine and immunosuppressants, and various TGT dosages. The results showed no statistical heterogeneity between trials (Supplementary Figures S1–S3).

### Secondary outcomes

Supplementary Figures S1–S6 present the evaluation of the secondary outcomes. Compared with the CTs alone, TGT plus CTs was associated with significant improvement in the levels of 24-h urine protein (*MD*_1m_ = −0.77, 95% *CI* = −1.34 to −0.20, *p* = 0.008; *MD*_3m_ = −0.68, 95% *CI* = −0.84 to −0.52, *p* < 0.00001; Supplementary Figure S4), anti-dsDNA (*MD*_3m_ = −3.32, 95% *CI* −4.80 to −1.83, *p* < 0.0001; Supplementary Figure S5), complement proteins (*MD*_C3–3m_ = 0.20, 95% *CI* 0.18 to 0.22, *p* < 0.00001; *MD*_C4–3m_ = 0.12, 95% *CI* 0.11 to 0.13, *p* < 0.00001; Supplementary Figure S6), and immunoglobulins (*MD*_IgG-3m_ = −2.26, 95% *CI* −3.36 to −1.16, *p* < 0.0001; Supplementary Figures S7). Compared with MTX, TGT was also associated with statistical reduction in the level of 24-h urine protein (*MD*_6m_ = −0.32, 95% *CI* = −0.45 to −0.19, *p* < 0.00001; Supplementary Figure S8). We found no statistical difference between two groups in the level of complement protein C3 (*MD*_6m_ = 0.02, 95% *CI* = −0.16 to 0.20, *p* = 0.83; Supplementary Figure S9).

### Safety outcomes

**FIGURE 6:**
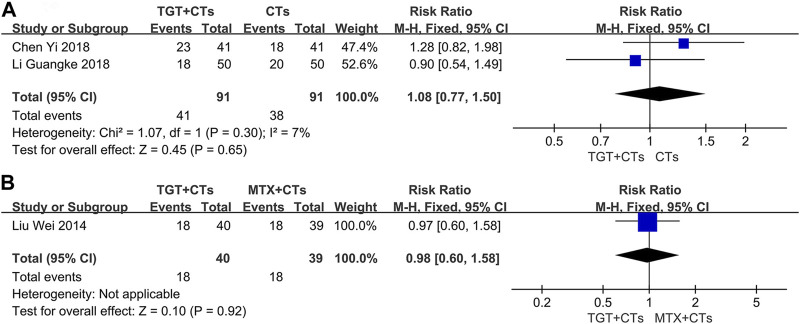
Forest plot of the incidence of adverse reactions (*TGT + CTs vs. CTs*) **(A)** and incidence of adverse reactions (*TGT + CTs vs. MTX + CTs*) **(B)**.

**FIGURE 7:**
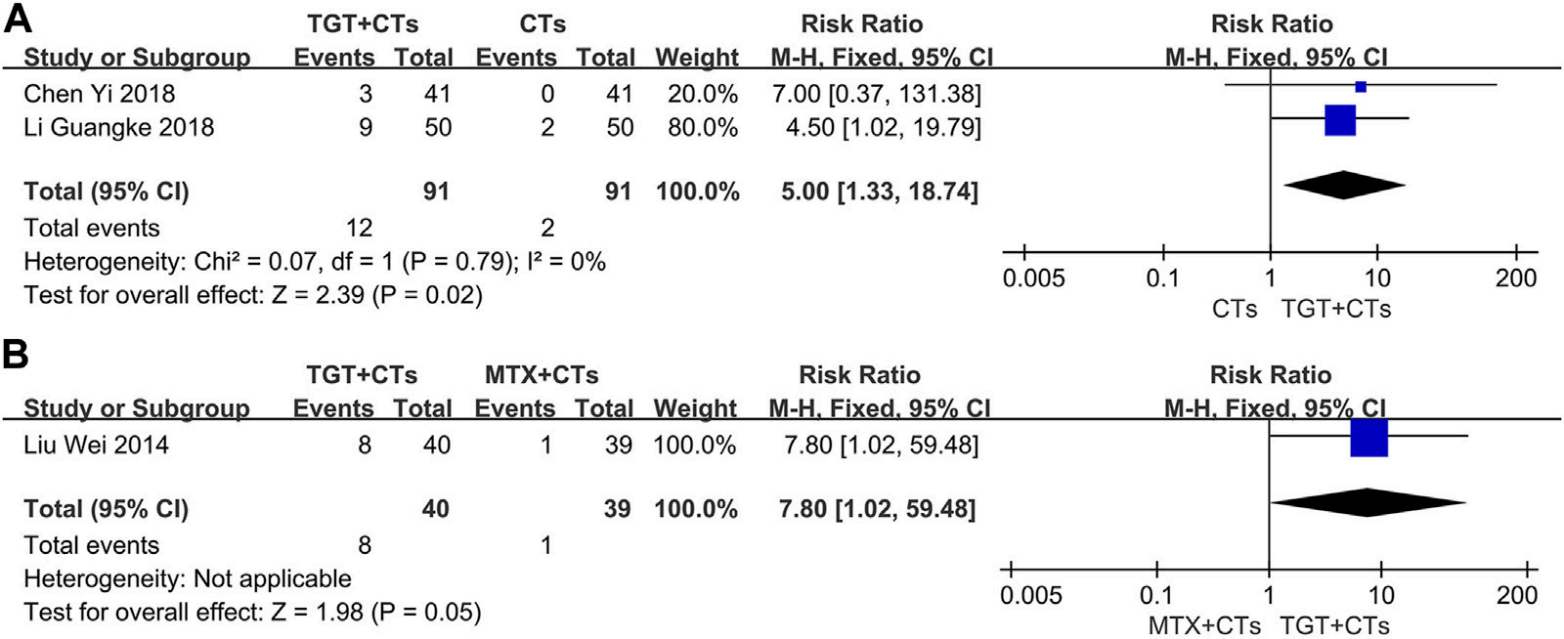
Forest plot of the incidence of menstrual disturbance (*TGT + CTs vs. CTs*) **(A)** and incidence of menstrual disturbance (*TGT + CTs vs. MTX + CTs*) **(B)**.

Three trials (Liu et al., 2014; Chen, 2018; Li et al., 2018) reported adverse reactions. No statistically significant difference in the incidence of all adverse reactions between the TGT groups and the control groups was observed (*RR*_TGT+CTs vs. CTs_ = 1.08, 95% *CI* = 0.77 to 1.50, *p* = 0.65; *RR*_TGT vs. MTX_ = 0.98, 95% *CI* = 0.60 to 1.58, *p* = 0.92; Figure 6). However, TGT was associated with a high risk of menstrual disturbance (*RR*_TGT+CTs vs. CTs_ = 5.00, 95% *CI* = 1.33 to 18.74, *p* = 0.02; *RR*_TGT vs. MTX_ = 7.80, 95% *CI* = 1.02 to 59.48, *p* = 0.05; Figure 7). The adverse effects in the TGT group also included abnormal liver function, respiratory tract infection, leukopenia, thrombocytopenia, nausea, and vomiting.

### Trial sequential analysis

**FIGURE 8:**
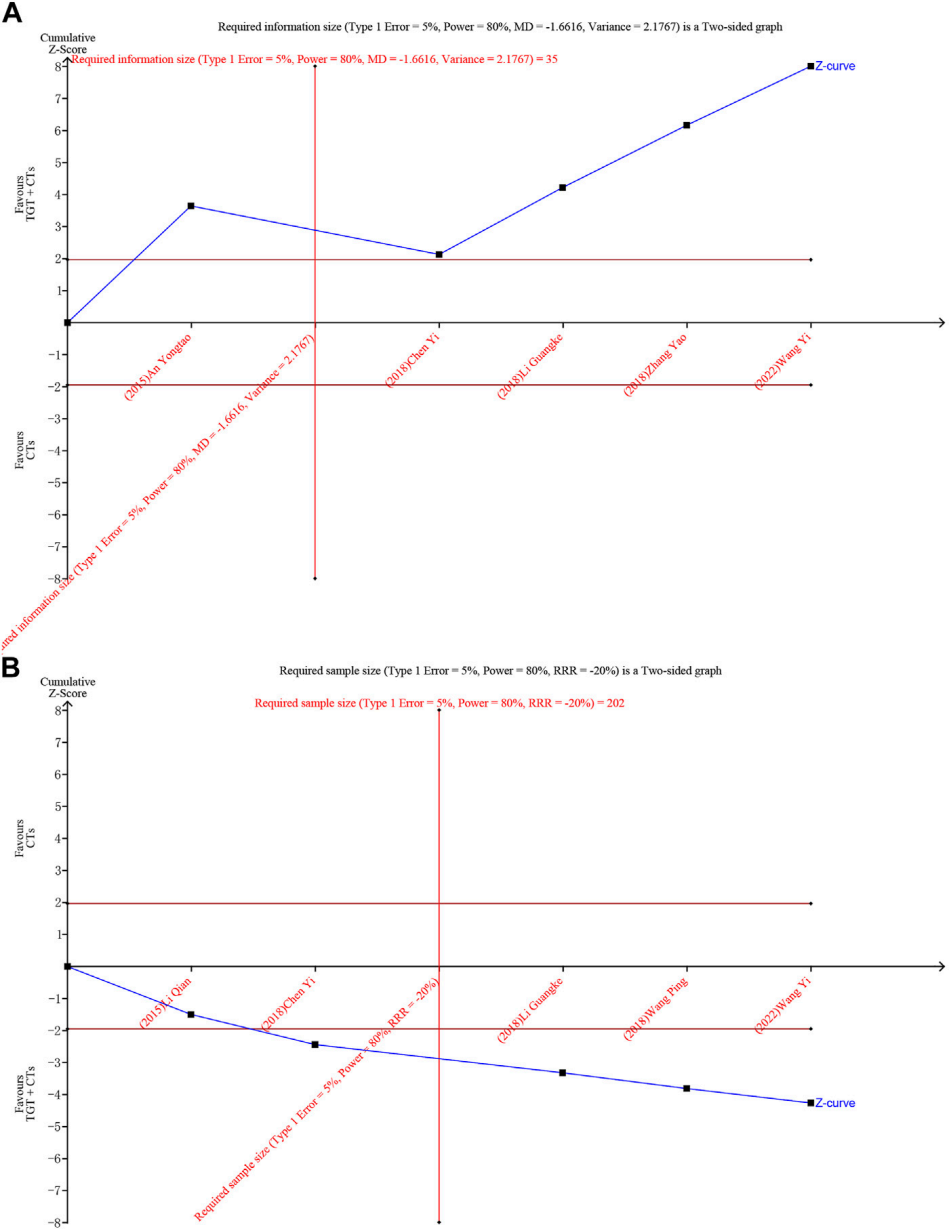
Trial sequential analysis of the SLEDAI score **(A)** and ORR **(B)**.

As for the SLEDAI score and ORR, we performed TSA to control for the risks of random errors. Type I error was of 5% and type II error was of 20%. TSA showed that the blue curves (cumulative Z-score) crossed the horizontal red lines (traditional boundaries of 5% significance) and vertical red lines (required information sizes) (Figure 8), indicating that the robustness of the results was confirmed.

### Publication bias

**FIGURE 9:**
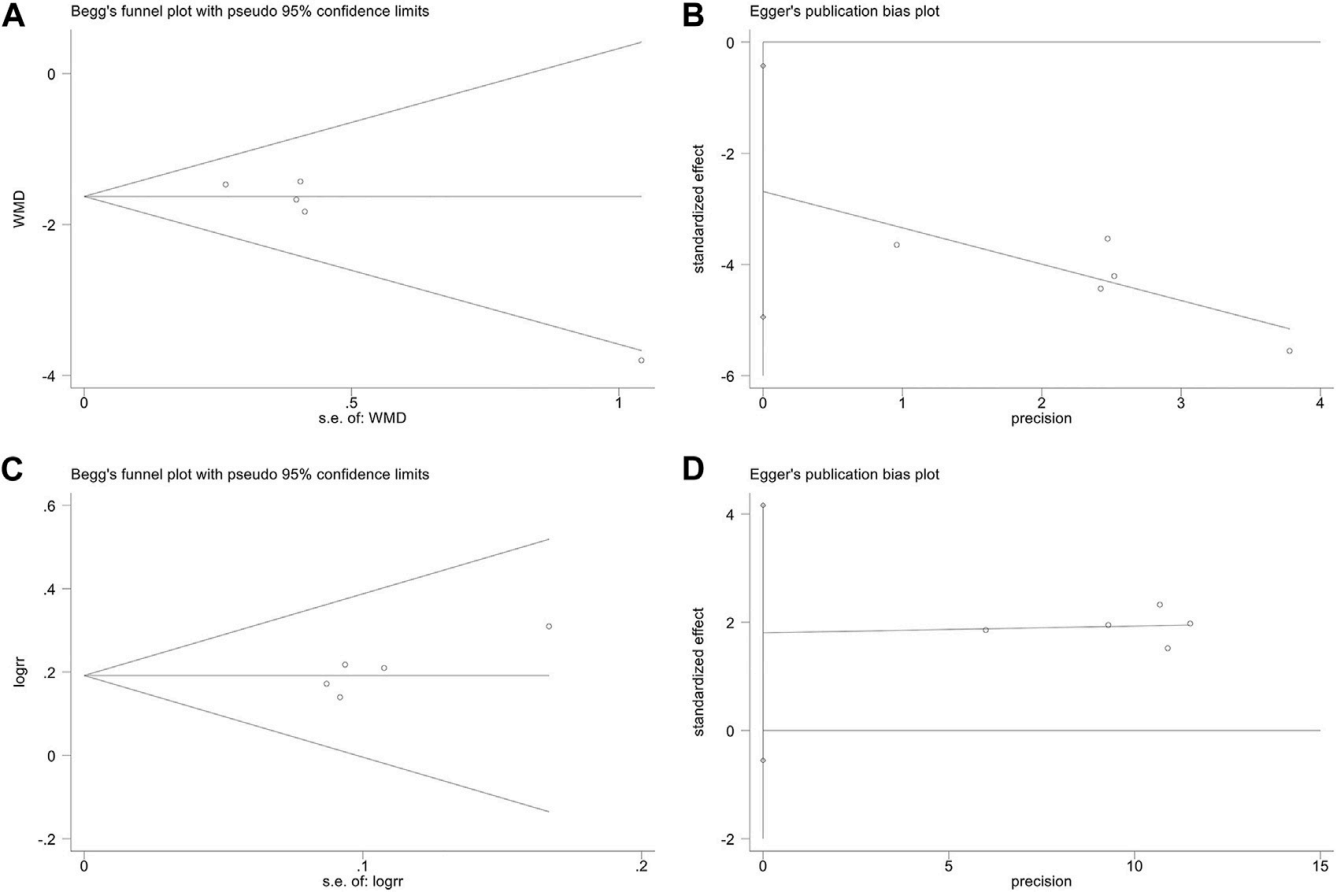
Publication bias. The Begg’s test on the SLEDAI score **(A)**. The Egger’s test on the SLEDAI score **(B)**. The Begg’s test on ORR **(C)**. The Egger’s test on ORR **(D)**.

To detect the publication bias for the SLEDAI score and ORR, Begg’s rank correlation and Egger’s linear regression tests were performed. The Begg’s rank correlation test (Figure 9A) of the SLEDAI score showed that the *p*-value was greater than 0.05 (*p* = 0.086), but the Egger’s linear regression test (Figure 9B) showed that the *p*-value was less than 0.05 (*p* = 0.032). Since Egger’s linear regression test is more accurate than Begg’s test, we suspected that the results of the SLEDAI score might be affected by publication bias. The Begg’s test (Figure 9C) and Egger’s linear regression test (Figure 9D) of the ORR illustrated that the *p*-value was all greater than 0.05 (Begg, *p* = 0.221; Egger, *p* = 0.093), which suggested no publication bias.

The potential publication bias might be caused by the following factors: small sample size effects; clinical heterogeneity in durations of treatment; and all the included RCTs are published in Chinese, leading to language publication bias.

### GRADE assessment

We chose six outcomes provided for the comparison of TGT plus CTs *versus* CTs alone, including SLEDAI, SLEDAI-3m, ORR, ORR-3m, incidence of adverse reactions, and incidence of menstrual disturbance. The certainty of the evidence was generally compromised by a high risk of bias, as none of the included trials guaranteed that the allocation concealment had been implemented and that the participants, investigators, or assessors had been blinded. For the outcomes from small samples, we downgraded the certainty by two levels due to a combination of high risk of bias and substantial imprecision. In addition, we downgraded the certainty of evidence on SLEDAI by one level due to potential publication bias detected by Egger’s linear regression test. The certainty of evidence was rated as moderate to low. An overview of the evidence certainty is shown in Table 2.

**TABLE 2:** GRADE summary of outcomes for TGT + CTs *versus* CTs for patients with SLE.

Outcomes	No. of participants (studies)	Anticipated Absolute effects (95% *CI*)		Relative effect (95% *CI*)	Certainty of the evidence (GRADE)
		Risk with CTs	Risk difference with TGT + CTs		
SLEDAI	292 (5)	The mean SLEDAI-1m ranged from 2 to 8.9	The mean SLEDAI in the TGT + CTs group was 1.66 lower (2.07 lower to 1.26 lower)	-	⊕⊕○○
					LOW^a^ ^,^ ^c^
SLEDAI-3m	112 (2)	The mean SLEDAI-3m ranged from 2 to 6.98	The mean SLEDAI-3m in the TGT + CTs group was 1.53 lower (1.96 lower to 1.1 lower)	-	⊕⊕○○
					LOW^a^ ^,^ ^b^
ORR	333 (5)	751 per 1,000	158 more per 1,000 (83 more to 240 more)	*RR* 1.21 (1.11–1.32)	⊕⊕⊕○
					MODERATE^a^
ORR-3m	249 (3)	774 per 1,000	147 more per 1,000 (54 more to 248 more)	*RR* 1.19 (1.07–1.32)	⊕⊕○○
					LOW^a^ ^,^ ^b^
Incidence of adverse reactions	182 (2)	418 per 1,000	33 more per 1,000 (96 fewer to 209 more)	*RR* 1.08 (0.77–1.50)	⊕⊕○○
					LOW^a^ ^,^ ^b^
Incidence of menstrual disturbance	182 (2)	22 per 1,000	88 more per 1,000 (7 more to 309 more)	*RR* 5.00 (1.33–18.74)	⊕⊕○○
					LOW^a^ ^,^ ^b^

CTs, conventional treatments; CI, confidence interval; ORR, overall response rate; RR, relative risks; TGT, Tripterygium Glycoside Tablets.

^a^ High risk of bias due to the unclear method of randomization, allocation concealment and blinding.

^b^ Substantial imprecision due to small sample sizes.

^c^ Potential publication bias detected by quantitative methods.

## Discussion

### Summary of findings

In this systematic review of eight RCTs with a total of 538 participants, the administration of TGT was significantly associated with decreased lupus activity, particularly in the first month of treatment, despite the methodological flaws and small samples in the original studies. In China, it is generally considered that the concentration- and time-dependent toxicity of TGT is associated with long-term use (Tong et al., 2004; Jiang et al., 2009). Therefore, the medication compliance of patients treated for three or 6 months may be worse than that of patients treated for 1 month, which provides a partial explanation for the most significant effect in the first month. In addition, the initial SLEDAI score of the trial with duration of 1 month was the highest, indicating that the effect of TGT may be related to the severity of lupus. Another important finding was that there was a trend toward an improved ORR, especially an improved significant response rate, of patients with lupus treated with TGT. Additionally, TGT were significantly associated with ameliorated surrogate outcomes, including improvement in the levels of urine protein, anti-dsDNA, IgG, C3, and C4. As a marker reflecting the degree of renal damage, 24-h urine protein is positively correlated with the lupus activity (Li et al., 2018). As characteristic antibodies of SLE, anti-dsDNA antibodies participate in the pathogenesis of SLE, and have even been explored as a target in the management of SLE (Wang and Xia, 2019). Fucosylation levels of anti-dsDNA IgG1 fluctuate with lupus activity (Han et al., 2022). Although complement proteins are non-specific immunological parameters, those that regularly decrease before the flare can be used to monitor lupus activity (Petri et al., 2013).

The high risk of menstrual disturbance indicated that TGT may be associated with a low risk of other adverse reactions (for example, gastrointestinal symptoms) despite insufficient data (on the premise that there was no significant difference in the overall incidence of adverse reactions between the TGT and control groups). Pregnant women and women of childbearing age with pregnancy requirements remain contraindications.

One trial showed that there was no significant difference between the TGT group and the MTX group in controlling lupus activity, which seems to be direct but weak evidence that TGT may be an alternative to MTX for patients with SLE with moderate to severe activity. A previous RCT during 24 weeks, conducted in Bangladesh, indicated that 10 mg of MTX administered weekly appeared to be as effective as 150 mg of chloroquine administered daily for SLE with an acceptable safety (Islam et al., 2012). Regarding the regimen of 60 mg TGT daily plus glucocorticoid, it remains unclear whether its toxicity is acceptable, especially considering the damage to the female reproductive system.

### Comparison with previous studies

A previous systematic review of *Tw*HF for SLE was selected as a conference abstract at the American College of Rheumatology/Association of Rheumatology Health Professionals Annual Scientific Meeting, 2015 (Ye et al., 2015). However, our systematic review remains imperative due to the following: 1) In the eligibility criteria of the previous systematic review, there was no strict limitation on the interventions, including a variety of *Tw*HF preparations and other TCMs, which led to the substantial clinical heterogeneity. 2) Limited by the format of conference abstract, nearly all critical items in the Assessment of Multiple Systematic Reviews 2 (AMSTAR 2) (Shea et al., 2017) and the PRISMA guidelines (Page et al., 2021) were not reported. 3) Although a protocol for systematic review and meta-analysis of *Tw*HF against SLE was published (Chen et al., 2020), no results have been found to date.

### Strengths and limitations

Our study has several important strengths. Firstly, to our knowledge, this is the first systematic review assessing the efficacy and safety of TGT for SLE rigorously in accordance with the PRISMA statement and reporting the level of evidence following the GRADE approach. Secondly, based on the specific efficacy criteria, we speculated that the patients with significant response might have similar clinical characteristics to the responders of SRI. According to the SRI (Luijten et al., 2012), a responder is defined as having ≥4 points reduction from baseline in Safety of Estrogens in Lupus Erythematosus National Assessment SLEDAI, no new severe disease activity, no more than one new moderate organ score, and no worsening in the physicians’ global assessment. The significant response positivity partially meets the above three criteria. Hence, we further performed an evaluation of significant response rate, which is a supplement to the insufficient evaluation of SRI-4. Thirdly, our interpretations of the effects on controlling lupus activity were based on an estimate of the minimal clinically significant differences. A study based on data from a large cohort of patients with SLE indicated that the difference in change of SLEDAI-2K score between patients requiring an increase in treatment and those without additional treatment is 2.6 (Yee et al., 2011). This estimated difference can be recommended as a basis for determining the minimal clinically important treatment effects. Our subgroup analysis, therefore, revealed that only the difference in SLEDAI score at 1 month exceeded the threshold of clinical significance.

However, our study also has several limitations. Firstly, few trials reported the version of SLEDAI. The original SLEDAI focuses on new onset or recurrence of rash, mucous membranes, alopecia, and proteinuria, whereas the SLEDAI-2K allows for persistent lupus activity. Secondly, we failed to judge the safety and acceptability of TGT based on the incidence of adverse reactions only. The description of adverse reactions lacked information regarding their severity, frequency, and correlation with TGT. Thirdly, considering that the included trials are all Chinese literature with small sample sizes, we failed to rule out publication biases. Finally, and most importantly, critical methodological flaws were present, including unclear allocation concealment and unblinded design; therefore, the selection and performance/detection bias might have weakened the inference of TGT effects.

## Implications for future research

Considering the aforementioned limitations, we put forward a few suggestions.

(1) For the careful evaluation of the safety profile, observing the long-term fertility function of patients with menstrual disorders will provide more mature evidence regarding the tolerance profile of TGT. Safety should take precedence over efficacy. And a systematic review conducted by the Peking University School of Public Health showed that the risk of reproductive toxicity induced by *Tw*HF was as high as 17.9% (Sun et al., 2014). In addition, researchers should fully report the hepatotoxicity induced by TGT, including the symptoms, serum transaminase levels, and changes after drug withdrawal. With the research on the modernization of TCMs, various Chinese botanical drugs have been shown to decrease the toxicity of TGT (Liu et al., 2019). Numerous studies have revealed the protective effect of Semen Cuscutae flavonoids *in vivo*, which improves the premature ovarian failure and spermatogenic cell damage caused by TGT (Ren et al., 2018; Zhang et al., 2019; Huang et al., 2020). In addition, total glucosides of paeony, a promising alternative therapy to prevent lupus flares (Chen et al., 2022), may be effective against the acute liver injury induced by TGT in mice (Zhou et al., 2007). Therefore, it is necessary to study the efficacy and safety of total glucosides of paeony plus TGT for the treatment of SLE.
(2) For the TGT production improvements, we recommend the standardization of the process to avoid uneven product quality. In addition to controlling the content of active compounds, recently emerging novel drug delivery carriers and the innovation of traditional oral dosage forms are expected to achieve the toxicity reduction. A variety of TP delivery systems, including nano, polymeric micelle, and microemulsion systems, have been developed to alleviate adverse effects and improve bioavailability (Xu et al., 2013; Wang et al., 2019; Ren et al., 2021).
(3) For the assessment of lupus activity, researchers are encouraged to use SLEDAI-2K for the measurement of persistent activity and SRI for a sensitive response analysis to obtain clinically meaningful changes simultaneously. The SLEDAI-2K has a limited ability to identify all clinically meaningful changes in lupus activity (Jesus et al., 2019), and therefore, the SRI, requiring full improvement in some manifestations, might be a more suitable choice.

Future trials should be designed and performed on the basis of rigorous methodology, including a calculated sample size, a long follow-up period, a pre-registered protocol, and a blinded method. In addition, results should be reported in accordance with the guidelines of SPIRIT-TCM Extension 2018 (Dai et al., 2019) and CONSORT-CHM Formulas 2017 (Cheng et al., 2017).

## Conclusion

Based on the low-certainty evidence, patients with SLE that have received TGT in addition to CTs (for example, glucocorticoids or combined with immunosuppressants) may experience an additional reduction in disease activity. Considering the inconclusive tolerability and previous reports that *Tw*HF-induced toxicity occurred frequently in cases of long-term administration, clinicians might consider short-term TGT (for example, 20 mg TGT 3 times daily for 1 month) as a complementary treatment for SLE, while monitoring lupus activity over time and adjusting glucocorticoid dosage accordingly.

## Data Availability

The original contributions presented in the study are included in the article/Supplementary Material, further inquiries can be directed to the corresponding authors.

## References

[B1] An Y. Fang X. (2015). Effects of tripterygium glycosides combined with glucocorticoid therapy on systemic lupus erythematosus and glucocorticoid receptor in mononuclear cell. Guangxi Med. J. 37, 620–622. 10.11675/j.issn.0253-4304.2015.05.11

[B2] Apostolopoulos D. Morand E. F. (2016). It hasn’t gone away: The problem of glucocorticoid use in lupus remains. Rheumatology 406, i114–i122. 10.1093/rheumatology/kew406 28013208

[B3] Astry B. Venkatesha S. H. Laurence A. Christensen-Quick A. Garzino-Demo A. Frieman M. B. (2015). Celastrol, a Chinese herbal compound, controls autoimmune inflammation by altering the balance of pathogenic and regulatory T cells in the target organ. Clin. Immunol. 157 (2), 228–238. 10.1016/j.clim.2015.01.011 25660987 PMC4410084

[B4] Balshem H. Helfand M. Schünemann H. J. Oxman A. D. Kunz R. Brozek J. (2011). GRADE guidelines: 3. Rating the quality of evidence. J. Clin. Epidemiol. 64 (4), 401–406. 10.1016/j.jclinepi.2010.07.015 21208779

[B5] Bérard A. Sheehy O. Zhao J. Vinet E. Quach C. Bernatsky S. (2021). Chloroquine and hydroxychloroquine use during pregnancy and the risk of adverse pregnancy outcomes using real-world evidence. Front. Pharmacol. 12, 722511. 10.3389/fphar.2021.722511 34408654 PMC8366774

[B6] Bombardier C. Gladman D. D. Urowitz M. B. Caron D. Chang C. H. Austin A. (1992). Derivation of the SLEDAI. A disease activity index for lupus patients. The Committee on Prognosis Studies in SLE. Arthritis & Rheumatism Official J. Am. Coll. Rheumatology. 35 (6), 630–640. 10.1002/art.1780350606 1599520

[B7] Chen F. Liu J. Zhao Z. Li Z. Wu K. (2020). Tripterygium and its plant extraction for systemic lupus erythematosus: A protocol for systematic review and meta analysis. Med. Baltim. 99 (34), e21909. 10.1097/MD.0000000000021909 PMC7447359 32846857

[B8] Chen Y. Wang L. Cao Y. Li N. (2022). Total glucosides of paeonia lactiflora for safely reducing disease activity in systemic lupus erythematosus: A systematic review and meta-analysis. Front. Pharmacol. 13, 834947. 10.3389/fphar.2022.834947 35173622 PMC8841895

[B9] Chen Y. Zeng L. Shu N. Jiang M. Wang H. Huang Y. (2018). Pestalotiopsis-like species causing gray blight disease on camellia sinensis in China. J. Kunming Med. Univ. 39, 98–106. 10.1094/PDIS-05-17-0642-RE 30673469

[B10] Cheng C. W. Wu T. X. Shang H. C. Li Y. P. Altman D. G. Moher D. (2017). CONSORT extension for Chinese herbal medicine Formulas 2017: Recommendations, explanation, and elaboration. Ann. Intern. Med. 167 (2), 112–121. 10.7326/M16-2977 28654980

[B11] Chinese Rheumatology Association. (2020). National clinical research center for dermatologic and immunologic diseases, and Chinese systemic lupus erythematosus treatment and research group. 2020 Chinese guidelines for the diagnosis and treatment of systemic lupus erythematosus. Chin. J. Intern Med. 59, 172–185. 10.3760/cma.j.issn.0578-1426.2020.03.002 32146743

[B12] Dai L. Chan K. K. Mao J. Tian Y. Gu J. Zhou J. (2020). Modified zhibai Dihuang pill, a traditional Chinese medicine formula, on steroid withdrawal in systemic lupus erythematosus: A systematic review and meta-analysis. J. Integr. Med. 18 (6), 478–491. 10.1016/j.joim.2020.08.007 32907784

[B13] Dai L. Cheng C. W. Tian R. Zhong L. L. Li Y. P. Lyu A. P. (2019). Standard protocol items for clinical trials with traditional Chinese medicine 2018: Recommendations, explanation and elaboration (SPIRIT-TCM extension 2018). Chin. J. Integr. Med. 25 (1), 71–79. 10.1007/s11655-018-2999-x 30484022

[B14] Doyno C. Sobieraj D. M. Baker W. L. (2021). Toxicity of chloroquine and hydroxychloroquine following therapeutic use or overdose. Clin. Toxicol. (Phila). 59 (1), 12–23. 10.1080/15563650.2020.1817479 32960100

[B15] Durcan L. O'Dwyer T. Petri M. (2019). Management strategies and future directions for systemic lupus erythematosus in adults. Lancet 393 (10188), 2332–2343. 10.1016/S0140-6736(19)30237-5 31180030

[B16] Fanouriakis A. Kostopoulou M. Alunno A. Aringer M. Bajema I. Boletis J. N. (2019). 2019 update of the EULAR recommendations for the management of systemic lupus erythematosus. Ann. Rheum. Dis. 78 (6), 736–745. 10.1136/annrheumdis-2019-215089 30926722

[B17] Ge J. C. Qian Q. Gao Y. H. Zhang Y. F. Li Y. X. Wang X. (2023). Toxic effects of Tripterygium glycoside tablets on the reproductive system of male rats by metabolomics, cytotoxicity, and molecular docking. Phytomedicine 114, 154813. 10.1016/j.phymed.2023.154813 37062137

[B18] Gladman D. D. Ibanez D. Urowitz M. B. (2002). Systemic lupus erythematosus disease activity index 2000. J. rheumatology 29 (2), 288–291. 11838846

[B19] Han J. Zhou Z. Zhang R. You Y. Guo Z. Huang J. (2022). Fucosylation of anti-dsDNA IgG1 correlates with disease activity of treatment-naïve systemic lupus erythematosus patients. EBioMedicine 77, 103883. 10.1016/j.ebiom.2022.103883 35182998 PMC8857559

[B20] Hochberg M. C. (1997). Updating the American College of Rheumatology revised criteria for the classification of systemic lupus erythematosus. Arthritis & Rheumatology. 40, 1725–25. 10.1002/art.1780400928 9324032

[B21] Huang C. He S. Guan Y. Zhou R. Tan N. Luo S. (2020). Evaluation of antiobesity activity of soybean meal products fermented by *Lactobacillus plantarum* FPS 2520 and *Bacillus subtilis* N1 in rats fed with high-fat diet. Chin. J. Clin. Pharmacol. 36, 667–675. 10.1089/jmf.2019.4643 32286891

[B22] Huybrechts K. F. Bateman B. T. Zhu Y. Straub L. Mogun H. Kim S. C. (2021). Hydroxychloroquine early in pregnancy and risk of birth defects. Am. J. Obstet. Gynecol. 224 (3), 290.e1–290.e22. 10.1016/j.ajog.2020.09.007 PMC7501839 32961123

[B23] Islam M. N. Hossain M. Haq S. A. Alam M. N. Ten K. P. Rasker J. J. (2012). Efficacy and safety of methotrexate in articular and cutaneous manifestations of systemic lupus erythematosus. Int. J. Rheum. Dis. 15 (1), 62–68. 10.1111/j.1756-185X.2011.01665.x 22324948

[B24] Jesus D. Rodrigues M. Matos A. Henriques C. Pereira Da Silva J. A. Inês L. S. (2019). Performance of SLEDAI-2K to detect a clinically meaningful change in SLE disease activity: A 36–month prospective cohort study of 334 patients. Lupus 28 (5), 607–612. 10.1177/0961203319836717 30895904

[B25] Jiang Y. Chen Q. Zhang Z. (2009). Analysis of adverse drug reaction of tripterygium glycosides tablets. West China Med. J. 24 (09), 2357–2359.

[B26] Jönsen A. Bengtsson A. A. Hjalte F. Petersson I. F. Willim M. Nived O. (2015). Total cost and cost predictors in systemic lupus erythematosus – 8-years follow-up of a Swedish inception cohort. Lupus 24 (12), 1248–1256. 10.1177/0961203315584812 25957301

[B27] Lazar S. Kahlenberg J. M. (2023). Systemic lupus erythematosus: New diagnostic and therapeutic approaches. Annu. Rev. Med. 74, 339–352. 10.1146/annurev-med-043021-032611 35804480

[B28] Li G. Li J. Yuan Y. Zhao Y. Wang G. (2018). Influence of tripterygium glycosides combined with prednisone on CD4+ CD25+ T cells in patients with systemic lupus erythematosus and its curative effect. Lab. Med. Clin. 15, 798–801. 10.3969/j.issn.1672-9455.2018.06.019

[B29] Li H. Guo F. Luo Y. Zhu J. Wang J. (2015). Efficacy of tripterygium glycosides tablet in treating ankylosing spondylitis: A systematic review and meta-analysis of randomized controlled trials. Clin. Rheumatol. 34 (11), 1831–1838. 10.1007/s10067-015-3043-6 26255190

[B30] Li M. Li J. Wang J. Li Y. Yang P. (2018). Serum level of anti-α-enolase antibody in untreated systemic lupus erythematosus patients correlates with 24-hour urine protein and D-dimer. Lupus 27 (1), 139–142. 10.1177/0961203317721752 28728510

[B31] Li Q. Luo Z. (2015). Efficacy of tripterygium glycosides combined with prednisone acetate in treatment of moderate activity type systemic lupus erythematosus. China Mod. Med. 22, 135–137.

[B32] Li X. Du F. Liu H. Ji J. Xing J. (2015). Investigation of the active components in Tripterygium wilfordii leading to its acute hepatotoxicty and nephrotoxicity. J. Ethnopharmacol. 162, 238–243. 10.1016/j.jep.2015.01.004 25582490

[B33] Li X. He Z. Ru L. Yuan Y. Yuan Z. Chen P. (2021). Efficacy and safety of Qinghao Biejia decoction in the treatment of systemic lupus erythematosus: A systematic review and meta-analysis. Front. Pharmacol. 12, 669269. 10.3389/fphar.2021.669269 34421590 PMC8378134

[B34] Li Y. Zhu W. He H. Bai L. Zhang L. Wang J. (2021). Efficacy and safety of tripterygium wilfordii Hook. F for connective tissue disease-associated interstitial lung disease: A systematic review and meta-analysis. Front. Pharmacol. 12, 691031. 10.3389/fphar.2021.691031 34177599 PMC8222720

[B35] Lin N. Zhang Y. Jiang Q. Liu W. Liu J. Huang Q. (2021). Clinical practice guideline for tripterygium glycosides/tripterygium wilfordii tablets in the treatment of rheumatoid arthritis. Front. Pharmacol. 11, 608703. 10.3389/fphar.2020.608703 33519474 PMC7845140

[B36] Lisnevskaia L. Murphy G. Isenberg D. (2014). Systemic lupus erythematosus. Lancet 384 (9957), 1878–1888. 10.1016/S0140-6736(14)60128-8 24881804

[B37] Liu B. Fan D. Shu H. He X. Lyu C. Lyu A. (2019). Transcriptome analysis of signaling pathways of human peritoneal mesothelial cells in response to different osmotic agents in a peritoneal dialysis solution. Chin. J. Exp. Traditional Med. Formulae 25, 181–190. 10.1186/s12882-019-1376-0 PMC6528310 31113397

[B38] Liu W. Yan L. Zhu Q. Shao F. (2014). Therapeutic effect of tripterygium glycosides plus prednisone on moderate active systemic lupus erythematosus. J. Chin. Pract. Diagn Ther. 28, 1234–1235. 10.13507/j.issn.1674-3474.2014.12.038

[B39] Liu Z. Li Z. Liu L. Mainolfi K. Chandrasekaran A. (2017). Effect of tripterygium glycoside on ovarian function loss in rats and interventional effect of total extract of China dodder seed: An experimental study. Hunan J. Tradit. Chin. Med. 33, 153–159. 10.1016/j.yrtph.2017.09.006

[B40] Luijten K. M. Tekstra J. Bijlsma J. W. Bijl M. (2012). The systemic lupus erythematosus responder index (SRI); a new SLE disease activity assessment. Autoimmun. Rev. 11 (5), 326–329. 10.1016/j.autrev.2011.06.011 21958603

[B41] Ma J. Dey M. Yang H. Poulev A. Pouleva R. Dorn R. (2007). Anti-inflammatory and immunosuppressive compounds from Tripterygium wilfordii. Phytochemistry 68 (8), 1172–1178. 10.1016/j.phytochem.2007.02.021 17399748

[B42] Mak A. Cheung M. W. L. Chiew H. J. Liu Y. Ho R. C. (2012). Global trend of survival and damage of systemic lupus erythematosus: Meta-analysis and meta-regression of observational studies from the 1950s to 2000s. Semin. Arthritis Rheu. 41 (6), 830–839. 10.1016/j.semarthrit.2011.11.002 22257558

[B43] Mu L. Hao Y. Fan Y. Huang H. Yang X. Xie A. (2018). Mortality and prognostic factors in Chinese patients with systemic lupus erythematosus. Lupus 27 (10), 1742–1752. 10.1177/0961203318789788 30060721

[B44] Mukwikwi E. R. Pineau C. A. Vinet E. Clarke A. E. Nashi E. Kalache F. (2020). Retinal complications in patients with systemic lupus erythematosus treated with antimalarial drugs. J. Rheumatol. 47 (4), 553–556. 10.3899/jrheum.181102 31474597

[B45] National Medical Products Administration of China (2003). National drug standards WS3-B-3350-98-2011. Beijing: National Medical Products Administration of China.

[B46] Page M. J. Mckenzie J. E. Bossuyt P. M. Boutron I. Hoffmann T. C. Mulrow C. D. (2021). The PRISMA 2020 statement: An updated guideline for reporting systematic reviews. BMJ n71, n71. 10.1136/bmj.n71 PMC8005924 33782057

[B47] Petri M. A. van Vollenhoven R. F. Buyon J. Levy R. A. Navarra S. V. Cervera R. (2013). Baseline predictors of systemic lupus erythematosus flares: Data from the combined placebo groups in the phase III belimumab trials. Arthritis Rheum. 65 (8), 2143–2153. 10.1002/art.37995 23754628

[B48] Piga M. Arnaud L. (2021). The main challenges in systemic lupus erythematosus: Where do we stand? J. Clin. Med. 10 (2), 243. 10.3390/jcm10020243 33440874 PMC7827672

[B49] Qiu Y. Hu N. Wen G. Deng Y. Zhang R. (2011). Effect of Tripterygium wilfordii polyglycosides on ovarian function in patients with systemic lupus erythematosus. Guangdong Med. 32, 3214–3215. 10.3969/j.issn.1001-9448.2011.24.024

[B50] Ren Q. Li M. Deng Y. Lu A. Lu J. (2021). Triptolide delivery: Nanotechnology-based carrier systems to enhance efficacy and limit toxicity. Pharmacol. Res. 165, 105377. 10.1016/j.phrs.2020.105377 33484817

[B51] Ren X. Zheng G. Su H. Zhang X. Zhai W. Tang J. (2018). Influence of flavonoids from Cuscutae Semen on cell cycle arrest, apoptosis and protein expression of spermatogenic cells induced by multi-glycoside from Tripterygium Wilfordii. Drug Eval. Res. 41, 55–60. 10.7501/j.issn.1674-6376.2018.01.009

[B52] Rengasamy P. (2017). Congenital malformations attributed to prenatal exposure to cyclophosphamide. Anti-Cancer Agents Med. Chem. Former. Curr. Med. Chemistry-Anti-Cancer Agents) 17 (9), 1211–1227. 10.2174/1871520616666161206150421 27924730

[B53] Sen M. Kurl A. Khosroshahi A. (2021). Pregnancy in patients with systemic lupus erythematosus after cyclophosphamide therapy. Lupus 30 (9), 1509–1514. 10.1177/09612033211021163 34053364

[B54] Shea B. J. Reeves B. C. Wells G. Thuku M. Hamel C. Moran J. (2017). Amstar 2: A critical appraisal tool for systematic reviews that include randomised or non-randomised studies of healthcare interventions, or both. BMJ 358, j4008. 10.1136/bmj.j4008 28935701 PMC5833365

[B55] Shen G. Zhuang X. Xiao W. Kong L. Tan Y. Li H. (2014). Role of CYP3A in regulating hepatic clearance and hepatotoxicity of triptolide in rat liver microsomes and sandwich-cultured hepatocytes. Food Chem. Toxicol. 71, 90–96. 10.1016/j.fct.2014.05.020 24910460

[B56] Sterne J. A. C. Savović J. Page M. J. Elbers R. G. Blencowe N. S. Boutron I. (2019). RoB 2: A revised tool for assessing risk of bias in randomised trials. BMJ 366, l4898. 10.1136/bmj.l4898 31462531

[B57] Sun F. Yang X. Ma D. Zhan S. (2014). Reproductive toxicity of tripterygium wilfordii Hook.f: A systematic review and meta-analysis. Chin. J. Pharmacovigil. 11, 94–99. 10.19803/j.1672-8629.2014.02.008

[B58] Thamer M. Hernán M. A. Zhang Y. I. Cotter D. Petri M. (2009). Prednisone, lupus activity, and permanent organ damage. J. rheumatology 36 (3), 560–564. 10.3899/jrheum.080828 PMC3624968 19208608

[B59] Tong J. Ma Y. Wu J. Chen J. Ni Q. Ding H. (2004). Study on the long-term toxicity and time rhythm of Tripterygium wilfordii. J. Chin. Med. Mater. 27 (12), 933–935. 10.3321/j.issn:1001-4454.2004.12.020

[B60] Tselios K. Gladman D. D. Touma Z. Su J. Anderson N. Urowitz M. B. (2019). Disease course patterns in systemic lupus erythematosus. Lupus 28 (1), 114–122. 10.1177/0961203318817132 30526328

[B61] Tunnicliffe D. J. Singh-Grewal D. Kim S. Craig J. C. Tong A. (2015). Diagnosis, monitoring, and treatment of systemic lupus erythematosus: A systematic review of clinical practice guidelines. Arthrit. Care Res. 67 (10), 1440–1452. 10.1002/acr.22591 25778500

[B62] Wang J. Wang C. Wu J. Li Y. Hu X. Wen J. (2019). Oral microemulsion based delivery system for reducing reproductive and kidney toxicity of Tripterygium glycosides. J. Microencapsul. 36 (6), 523–534. 10.1080/02652048.2019.1631402 31190589

[B63] Wang P. (2018). Clinical effect of tripterygium glycosides combined with hormone in the treatment of moderate active systemic lupus erythematosus. China Prac. Med. 13, 99–100. 10.14163/j.cnki.11-5547/r.2018.13.057

[B64] Wang X. Xia Y. (2019). Anti-double stranded DNA antibodies: Origin, pathogenicity, and targeted therapies. Front. Immunol. 10, 1667. 10.3389/fimmu.2019.01667 31379858 PMC6650533

[B65] Wang Y. (2022). Effect of tripterygium glycoside tablets in treatment of systemic lupus erythematosus. Liaoning J. Traditional Chin. Med. 49, 124–126. 10.13192/j.issn.1000-1719.2022.10.035

[B66] Wang Y. Han M. Pedigo C. E. Xie Z. Wang W. Liu J. (2021). Chinese herbal medicine for systemic lupus erythematosus: A systematic review and meta-analysis of randomized, placebo-controlled trials. Chin. J. Integr. Med. 27 (10), 778–787. 10.1007/s11655-021-3497-0 34319503

[B67] Wang Y. Jia L. Wu C. Y. (2008). Triptolide inhibits the differentiation of Th17 cells and suppresses collagen-induced arthritis. Scand. J. Immunol. 68 (4), 383–390. 10.1111/j.1365-3083.2008.02147.x 18782267

[B68] Xiao L. Xiao W. Zhan F. (2022). Targets of tripterygium glycosides in systemic lupus erythematosus treatment: A network-pharmacology study. Lupus 31, 319–329. 10.1177/09612033221076725 35067081

[B69] Xiong S. Li Y. Xiang Y. Peng N. Shen C. Cai Y. (2019). Dysregulation of lncRNA and circRNA expression in mouse testes after exposure to triptolide. Curr. Drug Metab. 20 (8), 665–673. 10.2174/1389200220666190729130020 31362668 PMC7062010

[B70] Xu L. Qiu Y. Xu H. Ao W. Lam W. Yang X. (2013). Acute and subacute toxicity studies on triptolide and triptolide-loaded polymeric micelles following intravenous administration in rodents. Food Chem. Toxicol. 57, 371–379. 10.1016/j.fct.2013.03.044 23583804

[B71] Xu Q. Zhang X. Ge S. Xu C. Lv Y. Shuai Z. (2023). Triptoquinone A and B exercise a therapeutic effect in systemic lupus erythematosus by regulating NLRC3. PeerJ 11, e15395. 10.7717/peerj.15395 37312878 PMC10259444

[B72] Ye Y. Chen B. Kalish R. A. Wang C. (2015). Tripterygium wilfordii for the treatment of systemic lupus systematosus: Meta-analysis of randomized controlled trials. Arthritis Rheum. 67. 10.1002/art.39448

[B73] Yee C. S. Farewell V. T. Isenberg D. A. Griffiths B. Teh L. S. Bruce I. N. (2011). The use of Systemic Lupus Erythematosus Disease Activity Index-2000 to define active disease and minimal clinically meaningful change based on data from a large cohort of systemic lupus erythematosus patients. Rheumatology 50 (5), 982–988. 10.1093/rheumatology/keq376 21245073 PMC3077910

[B74] Zhang B. Su H. Ren X. Li W. Ding Y. Zhang X. (2019). Study on mechanism of Cuscutae Semen flavonoids in improving reproductive damage of Tripterygium Glycosides Tablets in rats based on high-throughput transcriptome sequencing. China J. Chin. Mater Med. 44, 3478–3485. 10.19540/j.cnki.cjcmm.20190527.402 31602912

[B75] Zhang S. Li M. Zhang L. Wang Z. Wang Q. You H. (2021). Clinical features and outcomes of neuropsychiatric systemic lupus erythematosus in China. J. Immunol. Res. 2021, 1349042. 10.1155/2021/1349042 33532504 PMC7834780

[B76] Zhang Y. (2018). Effects of Tripterygium wilfordii improved on microcirculation dysfunction in patients with systemic lupus erythematosus. Guangxi: Guangxi University of Chinese Medicine. [master’s thesis].

[B77] Zhou Y. Zhang L. Liu W. (2007). Effect and mechanism of salvianolic acid B in attenuating elevated portal pressure in a rat model of portal hypertension induced by endothelin-1. Tianjin J. Traditional Chin. Med. 5, 61–64. 10.3736/jcim20070112 17214938

