# Supplementary material for: Tripterygium glycosides for safely controlling disease activity in systemic lupus erythematosus: a systematic review with meta-analysis and trial sequential analysis
Source: Front Pharmacol. 2023 Aug 4;14:1207385. doi: 10.3389/fphar.2023.1207385 (PMC10436586; doi:10.3389/fphar.2023.1207385)
Supplement: Supplementary file 1 [file DataSheet1.docx]

Supplementary Material

Tripterygium Glycosides for Safely Controlling Disease Activity in Systemic Lupus Erythematosus: A Systematic Review with Meta-Analysis and Trial Sequential Analysis

Yifan Chen^1^†, Liuding Wang^1^†, Nannan Li^1^*, Caiyun Zhou^1^*

^1^ Xiyuan Hospital, China Academy of Chinese Medical Sciences, Beijing 100091, China

*** Correspondence:**Nannan Li

lnn0648@126.com

Caiyun Zhou
zcy640111@126.com

# Supplementary Figures and Tables

## Supplementary Figures


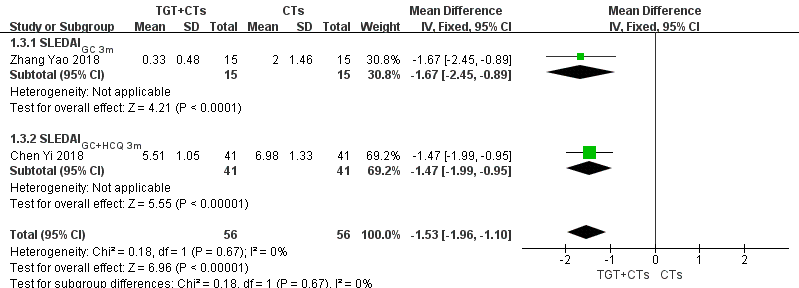


**Supplementary Figure 1.** The subgroup analysis of SLEDAI based on the CTs (*TGT+CTs vs. CTs*).


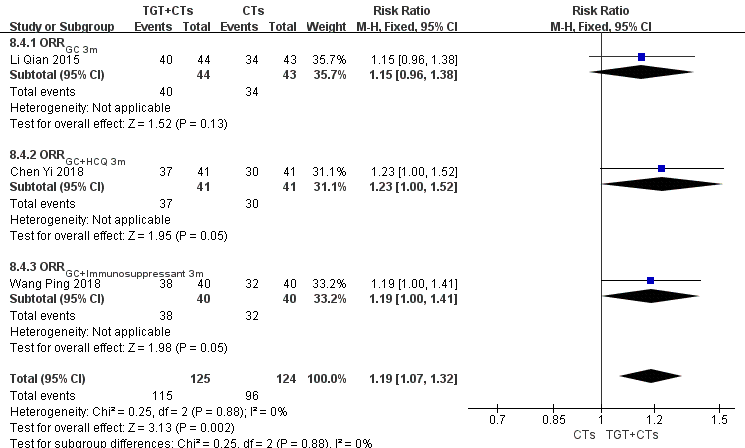


**Supplementary Figure 2.** The subgroup analysis of ORR based on the CTs (*TGT+CTs vs. CTs*).


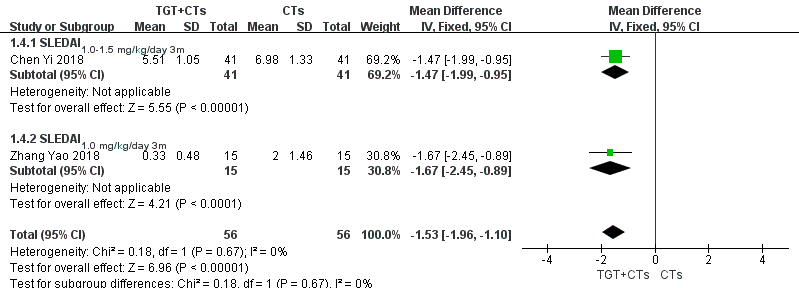


**Supplementary Figure 3.** The subgroup analysis of SLEDAI based on the dose of TGT (*TGT+CTs vs. CTs*).


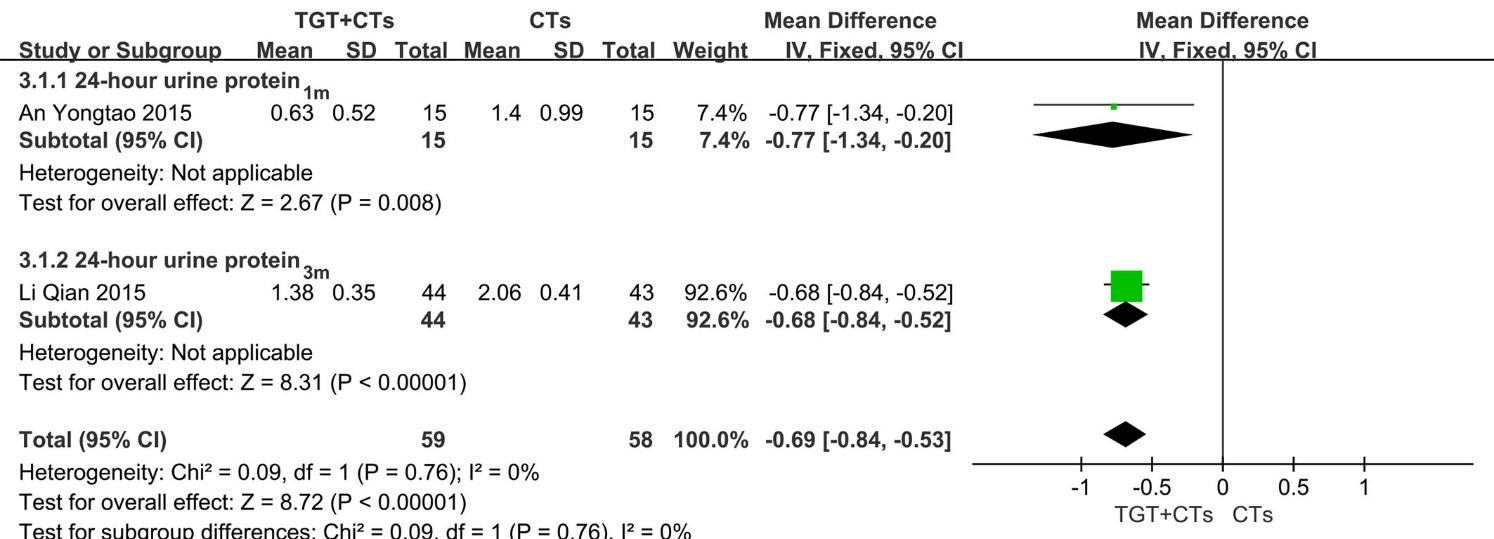


**Supplementary Figure 4.** Forest plot of 24-hour urine protein (*TGT+CTs vs. CTs*).


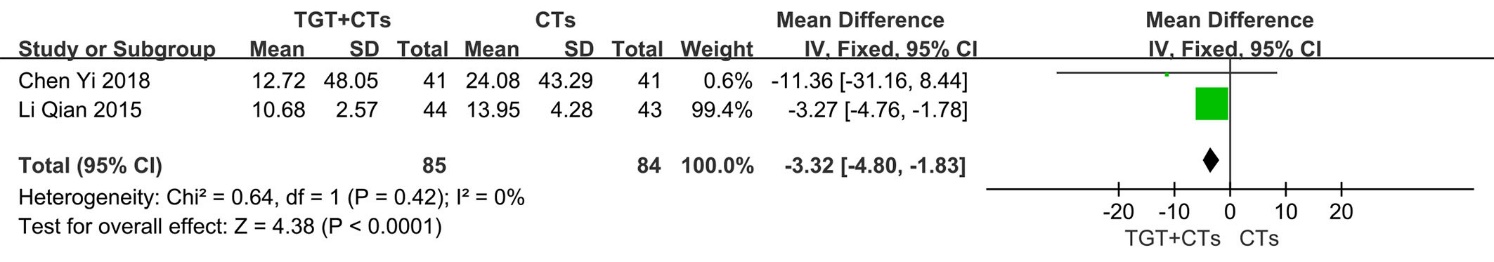


**Supplementary Figure 5.** Forest plot of anti-dsDNA (*TGT+CTs vs. CTs*).


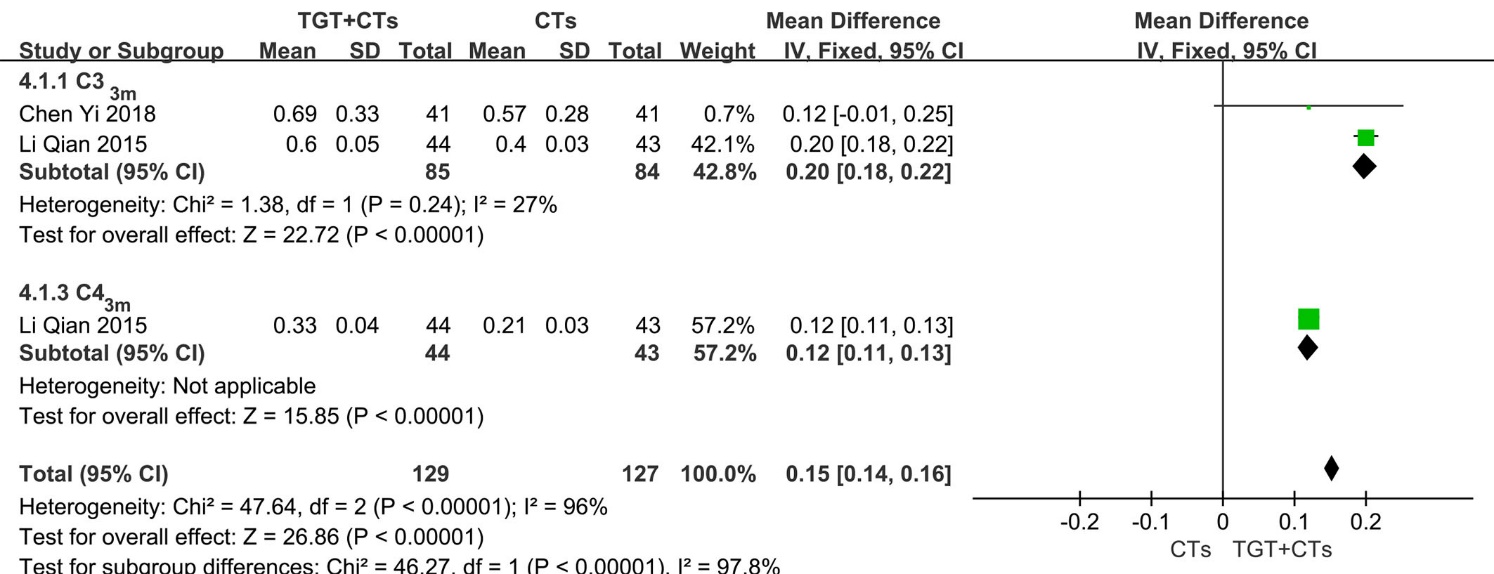


**Supplementary Figure 6.** Forest plot of complement proteins (*TGT+CTs vs. CTs*).

**
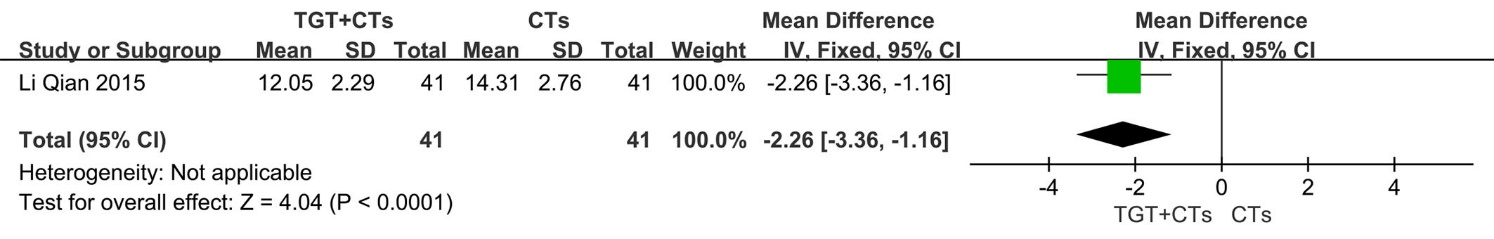
**

**Supplementary Figure 7.** Forest plot of IgG (*TGT+CTs vs. CTs*).

**
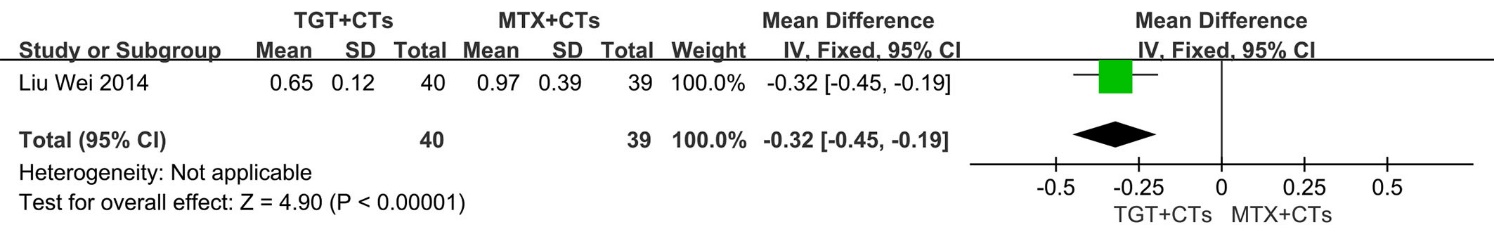
**

**Supplementary Figure 8.** Forest plot of 24-hour urine protein (*TGT+CTs vs. MTX+CTs*).


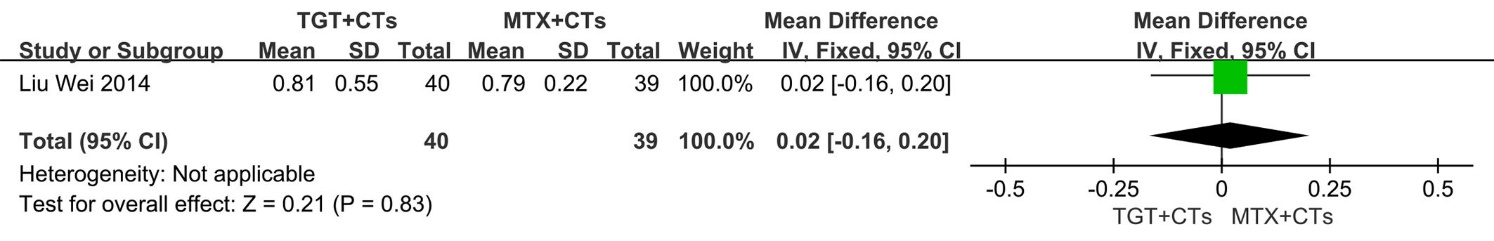


## Supplementary Figure 9. Forest plot of C3 (*TGT+CTs vs. MTX+CTs*).

## Supplementary Tables

**Supplementary Table 1.** The search strategies for all databases.

| **The search strategy for PubMed** | |
| --- | --- |
| **Number** | **Search terms** |
| #1 | Lupus Erythematosus, Systemic [MeSH Terms] |
| #2 | ((((Lupus Erythematosus, Systemic[Title/Abstract]) OR (Systemic Lupus Erythematosus[Title/Abstract])) OR (Lupus Erythematosus Disseminatus[Title/Abstract])) OR (lupus[Title/Abstract])) OR (sle[Title/Abstract]) |
| #3 | #1 OR #2 |
| #4 | Tripterygium [MeSH Terms] |
| #5 | (((((((((((((((Tripterygium[Title/Abstract]) OR (Tripterygiums[Title/Abstract])) OR (Tripterygium wilfordii[Title/Abstract])) OR (Tripterygium wilfordius[Title/Abstract])) OR (wilfordius, Tripterygium[Title/Abstract])) OR (Leigong Teng[Title/Abstract])) OR (Leigong Tengs[Title/Abstract])) OR (Teng, Leigong[Title/Abstract])) OR (Tengs, Leigong[Title/Abstract])) OR (Thundergod Vine[Title/Abstract])) OR (Thundergod Vines[Title/Abstract])) OR (Vine, Thundergod[Title/Abstract])) OR (Vines, Thundergod[Title/Abstract])) OR (Tripterygium hypoglaucum[Title/Abstract])) OR (Tripterygium hypoglaucums[Title/Abstract])) OR (hypoglaucums, Tripterygium[Title/Abstract]) |
| #6 | #4 OR #5 |
| #7 | #3 AND #6 |
| **The search strategy for Embase** | |
| **Number** | **Search terms** |
| #1 | exp systemic lupus erythematosus/ |
| #2 | exp lupus erythematosus/ |
| #3 | (lupus or sle).tw. |
| #4 | 1 or 2 or 3 |
| #5 | exp Tripterygium/ or exp Tripterygium wilfordii/ or exp Tripterygium wilfordii extract/ or exp Tripterygium hypoglaucum/ |
| #6 | Tripterygium.tw. |
| #7 | 5 or 6 |
| #8 | 4 and 7 |
| **The search strategy for Cochrane Library** | |
| **Number** | **Search terms** |
| #1 | MeSH descriptor: [Lupus Erythematosus, Systemic] explode all trees |
| #2 | (lupus):ti,ab,kw OR (sle):ti,ab,kw |
| #3 | MeSH descriptor: [Tripterygium] explode all trees |
| #4 | (Tripterygium):ti,ab,kw |
| #5 | #1 OR #2 |
| #6 | #3 OR #4 |
| #7 | #5 AND #6 |
| **The search strategy for CNKI** | |
| (SU = '系统性红斑狼疮' OR SU = '系统红斑狼疮' OR SU = '红蝴蝶疮') AND (SU= '雷公藤') | |
| **The search strategy for WanFang** | |
| 主题: ("系统性红斑狼疮" or "系统红斑狼疮" or "红蝴蝶疮") and 主题: ("雷公藤") | |
| **The search strategy for VIP** | |
| ((M=系统性红斑狼疮 OR 系统红斑狼疮 OR 红蝴蝶疮) OR (R=系统性红斑狼疮 OR 系统红斑狼疮 OR 红蝴蝶疮)) AND ((M=雷公藤) OR (R=雷公藤)) | |
| **The search strategy for CBM** | |
| #1 | "红斑狼疮, 系统性"[不加权:扩展] |
| #2 | "系统性红斑狼疮"[常用字段:智能] OR "系统红斑狼疮"[常用字段:智能] OR "红蝴蝶疮"[常用字段:智能] |
| #3 | "雷公藤"[不加权:扩展] |
| #4 | "雷公藤"[常用字段:智能] |
| #5 | #1 OR #2 |
| #6 | #3 OR #4 |
| #7 | #5 AND #6 |

**Supplementary Table 2.** The list of excluded studies.

| **Report excluded** | **Reason** |
| --- | --- |
| Wu Guoqin 2004 (1) | Improper intervention types |
| Bao Xiaohui 2003 (2) | Improper intervention types |
| Dai Lianyi 2001 (3) | Improper intervention types |

**References**

1. Wu G, Chen L. Viral antibody of systemic lupus erythematosus and treatment of Tripterygium wilfordii. The fourth national Tripterygium wilfordii Academic Conference: Shanghai, China 2004:476-477.
2. Bao X, Wu J, Zhou Z, Li L, Leng B, Wu Z. Comparative observation of curative effect of Chinese and Western Medicine on blood stasis syndrome of systemic lupus erythematosus. *Modern Journal of Integrated Traditional Chinese and Western Medicine* (2003) 12: 1129-1131. doi: 10.3969/j.issn.1008-8849.2003.11.005
3. Dai L. 30 cases of systemic lupus erythematosus treated with traditional Chinese Medicine. *Journal of traditional Chinese Medicine* (2001) 42: 284. doi: 10.3321/j.issn:1001-1668.2001.05.040

**Supplementary Table 3.** The source, quality control, and chemical characteristics of Tripterygium Glycosides Tablets.

| **Study** | **Species, source, concentration** | **Quality control reported**  **(Y/N)** | **Chemical characterisation of the preparation** |
| --- | --- | --- | --- |
| Liu et al., 2014 | Dried roots of *Tripterygium wilfordii* Hook.f., [Yuanda pharmaceutical Huangshi Feiyun Pharmaceutical Co., Ltd], concentration uncertainty | Y –National medicine permission number: Z42021212 | Tripterygium Glycosides Tablets  1. Triptolide:  Immunoregulatory, anti-inflammatory;  2. Wilforlide A:  anti-inflammatory, anti-tumor;  3. Triptonide:  anti-inflammatory, anti-tumor;  4. Tripterine:  anti-inflammatory, immunosuppressive, anti-tumor;  5. Wilforgine;  6. Wilforine.  (**HPLC**) |
| An and Fang, 2015 | Dried roots of *Tripterygium wilfordii* Hook.f., [Jiangsu Meitong Pharmaceutical Co.,Ltd], concentration uncertainty | Y –National medicine permission number: Z32021007 |  |
| Li and Luo, 2015 | Dried roots of *Tripterygium wilfordii* Hook.f., [Shanghai Fudan Fuhua Pharmaceutical Co., Ltd], concentration uncertainty | Y –National medicine permission number: Z31020415 |  |
| Chen, 2018 | Dried roots of *Tripterygium wilfordii* Hook.f., [Lunan Houpu Pharmaceutical Co., Ltd], concentration uncertainty | Y –National medicine permission number: Z37020344 |  |
| Li et al., 2018 | Dried roots of *Tripterygium wilfordii* Hook.f., [Yuanda pharmaceutical Huangshi Feiyun Pharmaceutical Co., Ltd], concentration uncertainty | Y –National medicine permission number: Z42021212 |  |
| Wang, 2018 | Dried roots of *Tripterygium wilfordii* Hook.f., [Shanghai Fudan Fuhua Pharmaceutical Co., Ltd], concentration uncertainty | Y –National medicine permission number: Z31020415 |  |
| Zhang, 2018 | Dried roots of *Paeonia lactiflora* Pall., [NA], concentration uncertainty | N |  |
| Wang, 2022 | Dried roots of *Paeonia lactiflora* Pall., [Zhejiang De'ende Pharmaceutical Co.,Ltd], concentration uncertainty | Y –National medicine permission number: Z33020422 |  |
